# Evaluation of anti-insulin receptor antibodies as potential novel therapies for human insulin receptoropathy using cell culture models

**DOI:** 10.1007/s00125-018-4606-2

**Published:** 2018-04-27

**Authors:** Gemma V. Brierley, Kenneth Siddle, Robert K. Semple

**Affiliations:** 1 University of Cambridge Metabolic Research Laboratories, Wellcome Trust-MRC Institute of Metabolic Science, Cambridge, UK; 2 National Institute for Health Research Cambridge Biomedical Research Centre, Addenbrooke’s Hospital, Cambridge, UK; 3 University of Edinburgh Centre for Cardiovascular Science, Queen’s Medical Research Institute, Little France Crescent, Edinburgh, EH16 4TJ UK

**Keywords:** Diabetes, Donohue syndrome, Insulin receptor, Insulin resistance, Insulin signalling, Monoclonal antibodies, Rabson–Mendenhall syndrome

## Abstract

**Aims/hypothesis:**

Bi-allelic loss-of-function mutations in the *INSR* gene (encoding the insulin receptor [INSR]) commonly cause extreme insulin resistance and early mortality. Therapeutic options are limited, but anti-INSR antibodies have been shown to activate two mutant receptors, S323L and F382V. This study evaluates four well-characterised murine anti-INSR monoclonal antibodies recognising distinct epitopes (83-7, 83-14, 18-44, 18-146) as surrogate agonists for potential targeted treatment of severe insulin resistance arising from insulin receptoropathies.

**Methods:**

Ten naturally occurring mutant human INSRs with defects affecting different aspects of receptor function were modelled and assessed for response to insulin and anti-INSR antibodies. A novel 3T3-L1 adipocyte model of insulin receptoropathy was generated, permitting conditional knockdown of endogenous mouse *Insr* by lentiviral expression of species-specific short hairpin (sh)RNAs with simultaneous expression of human mutant *INSR* transgenes.

**Results:**

All expressed mutant INSR bound to all antibodies tested. Eight mutants showed antibody-induced autophosphorylation, while co-treatment with antibody and insulin increased maximal phosphorylation compared with insulin alone. After knockdown of mouse *Insr* and expression of mutant INSR in 3T3-L1 adipocytes, two antibodies (83-7 and 83-14) activated signalling via protein kinase B (Akt) preferentially over signalling via extracellular signal-regulated kinase 1/2 (ERK1/2) for seven mutants. These antibodies stimulated glucose uptake via P193L, S323L, F382V and D707A mutant INSRs, with antibody response greater than insulin response for D707A.

**Conclusions/interpretation:**

Anti-INSR monoclonal antibodies can activate selected naturally occurring mutant human insulin receptors, bringing closer the prospect of novel therapy for severe insulin resistance caused by recessive mutations.

**Electronic supplementary material:**

The online version of this article (10.1007/s00125-018-4606-2) contains peer-reviewed but unedited supplementary material, which is available to authorised users.

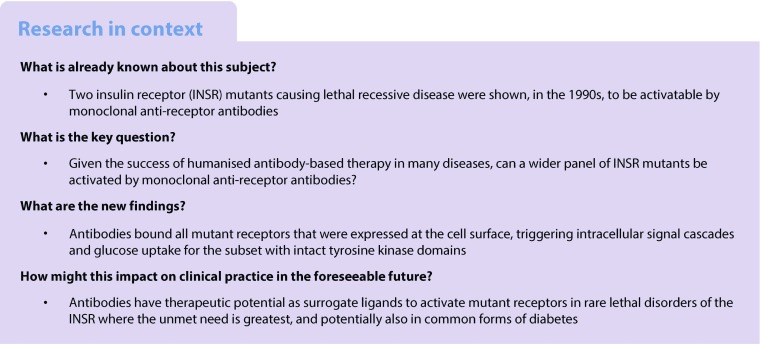



## Introduction

Insulin downregulates catabolic and activates anabolic pathways, suppresses apoptosis and promotes mitosis by activating a homodimeric receptor, tyrosine kinase [1, 2]. Human loss-of-function mutations in the *INSR* gene, which encodes the insulin receptor (INSR), were first reported in 1988 [3, 4]. Since then, more than 100 alleles causing severe insulin resistance have been described [5]. Bi-allelic *INSR* mutations produce extreme insulin resistance, clinically described as Donohue or Rabson–Mendenhall syndromes (OMIM #246200 or #262190). These also feature impaired linear growth and soft tissue overgrowth, with demise usually in the first 3 years of life in Donohue syndrome.

Some *INSR* mutations impair receptor processing and cell surface expression. Many mutations, however, are well expressed, but exhibit impaired insulin binding, impaired signal transduction, perturbed recycling kinetics or a combination of these [6]. Proof that the signalling defect of such mutant receptors might be circumvented by binding anti-receptor antibodies was provided for two mutations, one in a cell culture model and one as solubilised receptor [7, 8].

Therapeutic antibodies are now well established both in cancer, often blocking receptor signalling [9], and increasingly for non-cancer indications [10]. Interest in biological therapies targeting the INSR has recently rekindled, with inhibitory antibodies in Phase 1 human trials [11] and stimulatory antibodies shown to ameliorate diabetes in rodents [12–14] and primates [15]. Given the high clinical need in recessive insulin receptoropathy, we assessed the effect of monoclonal anti-INSR antibodies [16–20] on a series of disease-causing mutant INSRs.

## Methods

### Cell lines and culture conditions

Culture media for Chinese hamster ovary (CHO) Flp-In cells (Invitrogen, Carlsbad, CA, USA) and 3T3-L1 pre-adipocytes (Zenbio, Raleigh, NC, USA) are shown in electronic supplementary (ESM) Table 1. Cell lines were all mycoplasma negative by PCR. 3T3-L1 pre-adipocytes were grown to confluence and differentiation was induced by differentiation medium 1 for 72 h then differentiation medium 2 for a further 72 h. Adipocytes were maintained in adipocyte medium containing 1 μmol/l insulin ±1 μg/ml doxycycline (DOX). Experiments were undertaken at day 14 or 16 of differentiation.

### *hINSR* mutant expression constructs and generation of CHO Flp-In *hINSR* cells

Mutation numbering refers to mature *hINSR* ex11+ (GenBank M1005.1), which was amplified from pDNR-Dual (Clontech, Mountain View, CA, USA) using primers incorporating a C-terminus myc-tag. Sub-cloning is detailed in ESM Table 2. Mutations were generated with the Quickchange II XL kit (Stratagene, La Jolla, CA, USA). CHO Flp-In cells were transfected with pCDNA5/FRT/TO/*hINSR* and pOG44 using Lipofectamine 2000 (Invitrogen). The population surviving hygromycin B was used for experiments.

### Lentivirus production and infection of 3T3-L1 pre-adipocytes

Target sequences, primers, vectors and sub-cloning steps are detailed in ESM Table 3. Virus was packaged and concentrated as described by Shin et al [21]. 3T3-L1 pre-adipocytes were infected with the lowest multiplicity of infection (MOI) of virus needed to confer hygromycin B resistance. Several clones per line were characterised for endogenous *Insr* knockdown and adipocyte differentiation by Oil Red O staining [22]. For *hINSR* re-expression studies, 3T3-L1 murine *Insr*-knockdown (MmINSRKD) cells were infected with virus containing myc-tagged *hINSR* transgenes at the lowest MOI needed to confer G418 resistance to generate polyclonal populations. *hINSR* expression was confirmed by cDNA sequencing.

### Flow cytometry

CHO Flp-In *hINSR* cells were blocked by 5% (vol./vol.) FCS/FACS buffer (ESM Table 4) before incubation with primary antibodies for 1 h at 4°C. Bound antibodies were detected using FITC-conjugated anti-mouse IgG and a BD FACSCalibur Flow Cytometer (530 nm/30 nm bandwidth filter, Becton Dickinson, Franklin Lakes, NJ, USA). Stacked histograms were visualised with FCS Express 6 Plus (DeNovo Software, Glendale, CA, USA).

### Receptor autophosphorylation assays

CHO Flp-In *hINSR* cells were washed twice and serum starved (16 h) before stimulation with insulin, antibody or both for 10 min at 37°C/5% CO_2_ and lysed on ice in lysis buffer (ESM Table 4). Receptors were captured overnight at 4°C on anti-myc antibody 9E10-coated white Greiner Lumitrac 600 96 well plates. Phosphotyrosines on immunocaptured receptors were detected with biotin-conjugated 4G10 platinum phospho-tyrosine antibody and europium-labelled streptavidin. DELFIA enhancement solution was added and time-resolved fluorescence measured (excitation 340 nm/emission 615 nm).

### Downstream signal activation

3T3-L1 adipocytes were washed twice in DMEM, serum starved for 16 h in DMEM/0.5% BSA/1 μg/ml DOX and treated for 10 min at 37°C/5% CO_2_ with 10 nmol/l insulin, 10 nmol/l antibody or both in DMEM/0.5% (wt/vol.) BSA. Cells were washed, snap frozen and lysed on ice before centrifugation twice at 4°C for 15 min to pellet insoluble material and separate lipids prior to western blotting.

### Western blotting

Lysate, 10 μg, was resolved on NuPAGE 4–12% bis-tris gels or E-PAGE 48 8% gels (Life Technologies, Carlsbad, CA, USA) and transferred to nitrocellulose by iBlot (Life Technologies). Membranes were blocked in 3% BSA (wt/vol.)/tris-buffered saline with Tween 20 (TBST) before overnight incubation at 4°C with primary antibodies (ESM Table 5). Horseradish peroxidase (HRP)-conjugated secondary antibodies and Immobilon Western Chemiluminescent HRP substrate (Millipore, Darmstadt, Germany) were used to detect protein–antibody complexes, and grey-scale 16 bit tag image file formats (TIFFs) captured with an ImageQuant LAS4000 camera system (GE Healthcare Lifesciences, Marlborough, MA, USA). Each immunoblot in Fig. 4 and ESM Fig. 2 contained a sample of 3T3-L1 MmINSRKD *hINSR* wild type (WT) treated with 10 nmol/l insulin.

### Western blot image densitometry

Pixel density of grey-scale 16 bit TIFFs was determined in ImageJ 1.47v (NIH, Bethesda, MD, USA). The rectangle tool was used to select lanes and the line tool to enclose the peak of interest and subtract background. The magic-wand tool was used to select the peak area and obtain the raw densitometry value. Mean band intensities of total INSRβ, myc-tagged INSRβ, Akt, extracellular signal-regulated kinase 1/2 (ERK1/2), glycogen synthase kinase 3 (GSK3)α/β, ribosomal protein S6 kinase β 1 (p70S6K) and calnexin were used to normalise raw densitometry values for p-INSRβ, p-Akt, p-ERK1/2, p-GSK3α, p-p70S6K and p-Akt substrate of 160 kDa (p-AS160). Normalised values for phosphorylated targets were scaled to the mean WT INSR response to insulin.

### Glucose uptake

3T3-L1 adipocytes were washed twice (DMEM), serum starved for 16 h in low-glucose DMEM/0.2% BSA/1 μg/ml DOX, washed twice in PBS and then stimulated for 30 min at 37°C/5% CO_2_ with 10 nmol/l insulin, 10 nmol/l antibody or both in KRPH/0.2% BSA buffer (ESM Table 4). Cells were incubated with 1 mmol/l 2-deoxy-d-glucose for 5 min at 37°C/5% CO_2_ before washing (PBS), lysing with 0.1 mol/l NaOH, and snap freezing. Glucose uptake was measured by the fluorescence method of Yamamoto et al [23].

### Statistical analysis

One-way ANOVAs with Tukey’s multiple comparisons test were performed with GraphPad Prism 6 (GraphPad software, San Diego, CA, USA). Error bars represent SEM or SD as indicated. All experiments were performed at least three times.

## Results

### Assessment of mutant INSR cell surface expression and antibody binding

**Fig. 1:**
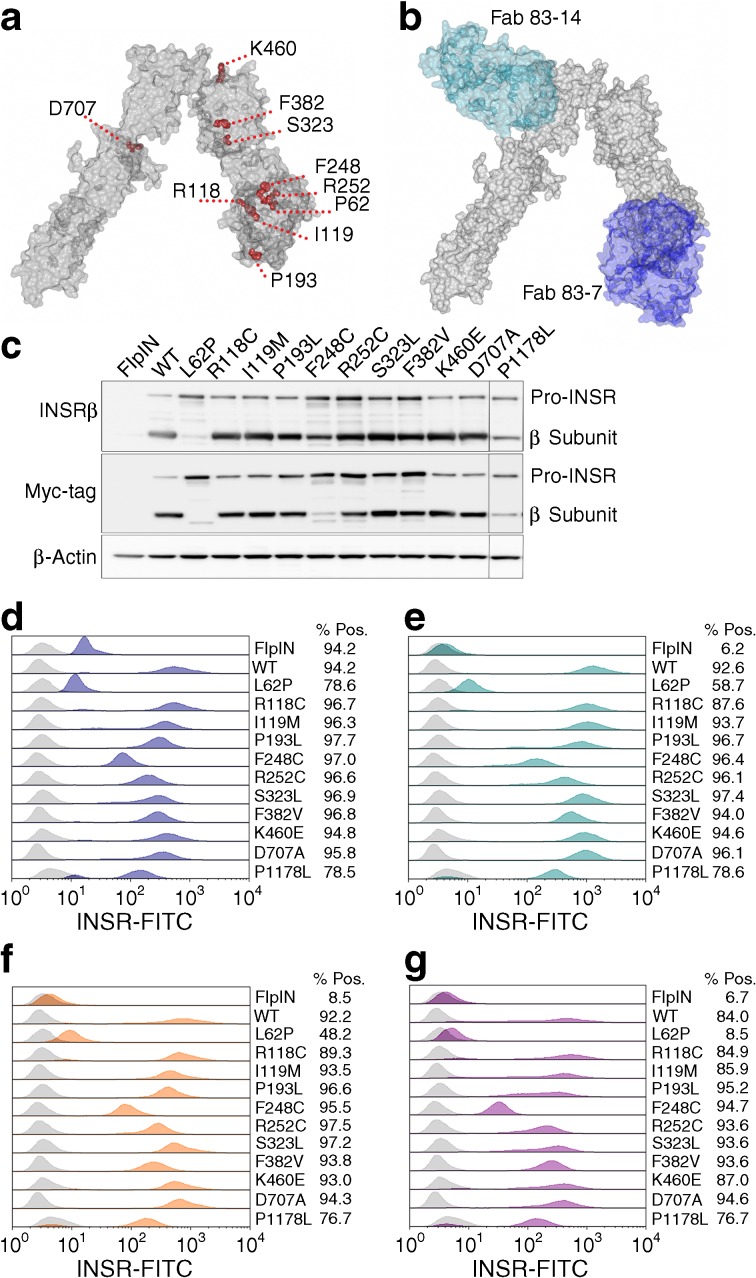
Mutant INSR is expressed at the cell surface and bound by anti-INSR antibody. (**a**) INSR monomer Protein Data Bank (PDB) structure entry 4ZXB [22] visualised with CCP4MG (v. 2.10.6); locations of mutated residues (this study) are highlighted in red. (**b**) INSR monomer in complex with Fab fragments 83-7 and 83-14, PDB structure 4ZXB [22]. (**c**) Western blot of lysates from CHO Flp-In cells stably expressing human WT or mutant INSR, as indicated. In INSRβ subunit and myc-tag blots, upper bands are pro-INSR and lower bands are mature processed β subunits, as indicated. (**d**–**g**) Stacked overlay single parameter histograms showing cell surface expression of INSR mutants bound by antibodies 83-7 (**d**), 83-14 (**e**), 18-44 (**f**) and 18-146 (**g**), as determined by flow cytometry. Intensity of INSR-FITC fluorescence is shown on the *x*-axis and the peak height indicates relative number of events. Isotype control IgG (light grey) was used as a negative control to generate a negative gate to determine the percentage of the population positive for anti-INSR antibody binding. Rightward shift of the peak (blue 83-7, cyan 83-14, orange 18-44, purple 18-146) from the IgG control is a function of both mutant INSR expression and antibody affinity. Pos, positive

Eleven INSR mutations were selected for study (ESM Table 6). Eight were chosen based on evidence of cell surface expression, prioritising mutations identified in multiple reports to maximise potential availability of participants for future trials. A previously unpublished mutation, F248C, that we identified in a child with Rabson–Mendenhall syndrome, was included opportunistically. The well-studied P1178L tyrosine kinase mutation [24, 25] and the L62P mutation, which severely impairs processing [26], were added as controls. Figure 1a displays the extracellular INSR mutations mapped onto the crystal structure of the INSR [27]. Four mouse monoclonal anti-human INSR antibodies were used, which had all previously been shown to have partial agonist activity at WT receptors, but different effects on kinetics and affinity of insulin binding (Table 1). As Fab fragments of 83-7 and 83-14 were used in determining the crystal structure of insulin-bound INSR [28], their binding epitopes are known (Fig. 1b).

**Table 1:** Characteristics of INSR antibodies studied

Characteristic	Antibody			
	83-7	83-14	18-44	18-146
Type	Mm mAb IgG1 (bivalent) [17]	Mm mAb IgG2a (bivalent) [17]	Mm mAb IgG2b (bivalent) [17]	Mm mAb IgG1 (bivalent) [17]
Epitope	CR domain^a^ [28]	Fn^1^ domain^a^ [28]	Within extracellular β subunit [17]	Within α subunit
Effect on INSR				
Insulin binding	Modestly enhances [17, 19]	Inhibits [17]	Modestly inhibits [17]	Enhances [17]
Pre-bound insulin	No effect on dissociation [18]	Inhibits dissociation [18]	No effect on dissociation [18]	Inhibits dissociation [18]
Autophosphorylation	Stimulates [19]	Stimulates [16]	Stimulates [19]	Not assessed
Kinase activity	Stimulates [18, 19]	Stimulates [18, 19]	Stimulates [18, 19]	Stimulates [18]
Biological outcomes				
Glucose uptake	Stimulates [16]	Stimulates [16]	Stimulates [16]	Not assessed
Lipogenesis	Stimulates [20]	Stimulates [20]	Stimulates [20]	Not assessed

Type, epitope and effect on WT INSR insulin binding, pre-bound insulin, autophosphorylation, kinase activity, glucose uptake and lipogenesis of murine monoclonal antibodies used throughout this study

^a^Precise epitope known

CR, cysteine-rich domain; Fn^1^, first fibronectin type III domain; mAb, monoclonal antibody; Mm, murine

Mutations were introduced into the B isoform of the INSR, believed to be the more important isoform for the metabolic actions of insulin [29]. To enable discrimination of endogenous INSR and human INSR mutants, a C-terminal myc-tag was used in the mutant constructs. Tagged mutants were expressed in CHO cells using the Flp-In system, ensuring differences in protein expression are due to differential processing or stability of receptor protein rather than differential mRNA expression. The mutants were well processed to mature β subunits, with the exception of L62P, for which β subunit was barely detectable. More modest reductions were seen for the previously unstudied F248C and for the P1178L mutation (Fig. 1c).

Cell surface expression and antibody binding of mutant INSR was assessed by flow cytometry (Fig. 1d–g). All INSR antibodies bound each mutant INSR, as shown by right-shifted peaks relative to control IgG, indicating no gross changes in receptor morphology. Poor expression of L62P was in keeping with prior reports [30], and L62P was not studied further. The rightward shift for mutants corresponded to expression of mature β subunits seen by immunoblotting, suggesting that relative shifts reflected differences in receptor expression rather than antibody affinities. Although some mutations are close to the epitope for antibody 83-7, none of the affected residues provides critical antibody contacts. Indeed, no difference in binding of 83-7 (Fig. 1d) to the mutant panel was seen compared with 83-14 (Fig. 1e), which binds to a surface unaffected by the mutations (Fig. 1a, b). Antibody 83-7 demonstrated cross-reactivity with endogenous CHO INSR, as evidenced by positive staining of CHO Flp-In parent cells, while the other antibodies did not detectably cross-react.

### Assessment of mutant INSR autophosphorylation in response to antibody and/or insulin

**Fig. 2:**
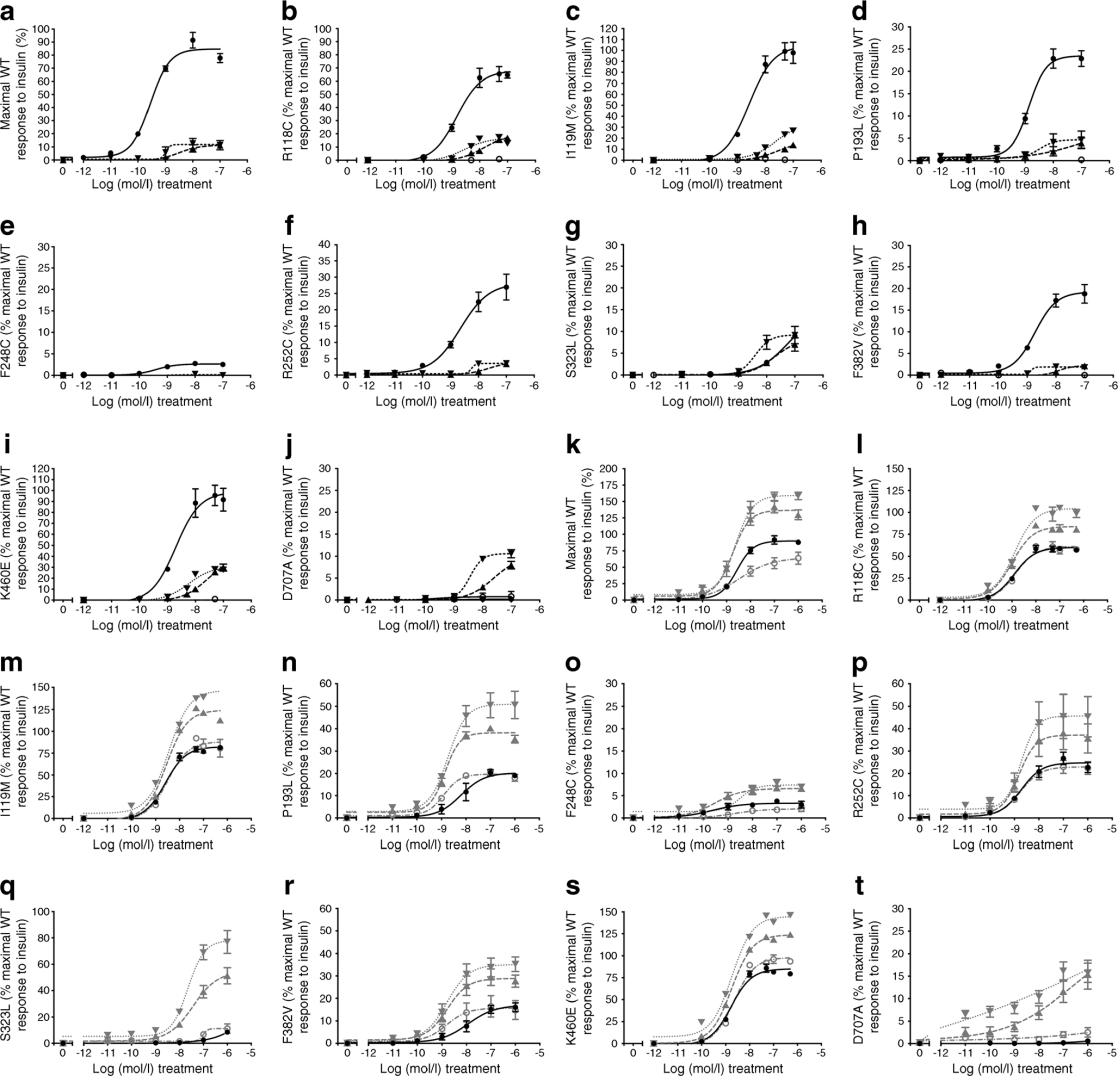
Insulin- and antibody-stimulated autophosphorylation of WT and mutant INSR. CHO Flp-In cells stably expressing either human WT or mutant INSR (as indicated) were serum starved prior to 10 min stimulation with increasing concentrations of insulin, antibody (83-7, 83-14) or control IgG (black lines), or increasing concentrations of insulin in the presence of 10 nmol/l antibody (grey lines). Cells were lysed and myc-tagged receptors were immunocaptured on 96 well plates and then incubated with biotin-conjugated 4G10 platinum antibody to detect phosphorylated tyrosine residues. Europium-labelled streptavidin was used to detect bound anti-phosphotyrosine antibody 4G10 by time-resolved fluorescence. The data points are the mean ± SEM of duplicate samples from three independent experiments and plotted on a log_10_ scale for the *x*-axis. Error bars are shown when larger than size of the symbols. In (**a–j**), single treatments are shown as follows: black circles (solid line), insulin; black up-pointing triangle (dashed line), 83-7; black down-pointing triangle (dotted line), 83-14; black open circles (solid line), control IgG. In (**k–t**), dual treatments are denoted by: grey up-pointing triangle (dashed line), insulin +10 nmol/l 83-7; grey down-pointing triangle (dotted line), insulin +10 nmol/l 83-14; grey circles (dotted/dashed line), insulin +10 nmol/l control IgG. pEC_50_ values are presented in Table 2

Trans-autophosphorylation of tyrosines in the intracellular INSR is the first detectable signalling event after insulin binding, so the ability of insulin and antibodies to induce tyrosine phosphorylation of mutant INSR was next examined using anti-myc immunoprecipitation and europium-based immunoassay. Most mutant receptors (P193L, F248C, R252C, S323L, F382V, D707A, P1178L) demonstrated diminished maximal autophosphorylation response to insulin, ranging from 0 to 27% WT (Fig. 2a–j, Table 2, data not shown for non-responsive P1178L). However, R118C, I119M and K460E showed autophosphorylation comparable with WT, and so were not studied further. Altered insulin EC_50_ was discernible only for S323L (Table 2), although the insulin concentration range tested and the small magnitude of responses precluded precise determinations.

**Table 2:** Autophosphorylation of WT and mutant INSR stimulated by insulin, antibody or insulin +10 nmol/l antibody

INSR/stimulation	Insulin	83-7	83-14	IgG	Insulin + 83-7	Insulin + 83-14	Insulin + IgG
WT							
EC_50_ (nmol/l)	0.3	3.0	–	–	1.8	2.3	3.4
pEC_50_	9.5	8.5	–	–	8.7	8.6	8.5
95% CI	9.7, 9.4	9.1, 7.9	–	–	8.9, 8.5	8.8, 8.5	9.0, 7.9
E_max_ (% Ins)	100	11	12	0	142	158	64
R118C							
EC_50_ (nmol/l)	1.4	13.2	2.6	–	1.0	1.3	1.4
pEC_50_	8.9	7.9	8.6	–	9.0	8.9	8.9
95% CI	9.1, 8.6	8.1, 7.6	9.0, 8.1	–	9.2, 8.8	9.1, 8.6	9.1, 8.6
E_max_ (% Ins)	100	26	26	1	131	160	94
E_max_ (% WT Ins)	65	17	17	0	85	104	61
I119M							
EC_50_ (nmol/l)	2.6	>25	>23	–	2.8	3.5	3.1
pEC_50_	8.6	>7.5	>7.6	–	8.6	8.5	8.5
95% CI	8.9, 8.3	–	–	–	8.7, 8.4	8.6, 8.3	8.8, 8.2
E_max_ (% Ins)	100	14	27	1	128	152	93
E_max_ (% WT Ins)	99	14	27	1	127	150	92
P193L							
EC_50_ (nmol/l)	1.4	>193	3.2	–	1.4	1.8	1.2
pEC_50_	8.9	>6.7	8.5	–	8.9	8.7	8.9
95% CI	9.1, 8.7	–	9.6, 7.3	–	9.0, 8.7	9.0, 8.5	9.1, 8.5
E_max_ (% Ins)	100	17	20	1	173	217	91
E_max_ (% WT Ins)	23	4	5	0	40	50	21
F248C							
EC_50_ (nmol/l)	0.4	–	–	–	0.3	3.2	1.8
pEC_50_	>7.2	–	–	–	9.5	8.5	8.7
95% CI	9.8, 9.0	–	–	–	10.0, 9.1	9.0, 8.0	9.9, 7.6
E_max_ (% Ins)	100	0	12	5	243	287	75
E_max_ (% WT Ins)	3	0	0	1	7	8	2
R252C							
EC_50_ (nmol/l)	2.2	–	–	–	1.6	1.8	1.7
pEC_50_	8.6	–	–	–	8.8	8.7	8.8
95% CI	9.0, 8.3	–	–	–	9.2, 8.4	9.3, 8.2	9.1, 8.4
E_max_ (% Ins)	100	15	15	1	141	166	85
E_max_ (% WT Ins)	27	4	4	0	38	45	23
S323L							
EC_50_ (nmol/l)	>58	18.6	3.9	–	36.4	22.4	>97
pEC_50_	>7.2	7.7	8.4	–	7.4	7.6	>7.0
95% CI	–	8.9, 6.5	8.8, 8.0	–	7.8, 7.1	7.9, 7.4	–
E_max_ (% Ins)	100	79	102	2	566	855	122
E_max_ (% WT Ins)	9	7	9	0	51	77	11
F382V							
EC_50_ (nmol/l)	1.8	12.3	–	–	1.6	1.6	1.5
pEC_50_	8.7	7.9	–	–	8.8	8.8	8.8
95% CI	9.0, 8.5	8.6, 7.2	–	–	9.2, 8.4	9.2, 8.4	9.6, 8.0
E_max_ (% Ins)	100	10	10	5	152	184	84
E_max_ (% WT Ins)	19	2	2	1	29	35	16
K460E							
EC_50_ (nmol/l)	1.9	25.2	5.7	–	1.7	1.8	2.2
pEC_50_	8.7	7.6	8.2	–	8.8	8.7	8.7
95% CI	9.1, 8.4	7.8, 7.2	8.5, 7.9	–	8.8, 8.7	8.9, 8.6	8.8, 8.5
E_max_ (% Ins)	100	31	29	1	129	152	100
E_max_ (% WT Ins)	96	30	28	1	124	146	96
D707A							
EC_50_ (nmol/l)	–	>24	3.6	–	–	–	–
pEC_50_	–	>7.6	8.4	–	–	–	–
95% CI	–	–	8.6, 8.2	–	–	–	–
E_max_ (% Ins)	100	4016	5383	416	7793	8230	1270
E_max_ (% WT Ins)	0	8	11	1	15	16	2

EC_50_, half-maximal effective concentration in nmol/l; pEC_50_, negative log of EC_50_ half-maximal effective concentration value in mol/l; 95% CI, 95% CI for pEC_50_; E_max_, maximum efficacy expressed as a % of a particular receptor response to insulin (% Ins) or as % WT receptor response to insulin (% WT Ins); Ins, insulin; −, not able to be determined

Antibodies 83-7 and 83-14 alone also elicited autophosphorylation of WT and all mutant INSRs except F248C and P1178L. In most instances, antibody response was lower than insulin response (Fig. 2a–j, Table 2); however, for S323L the maximal autophosphorylation response to 83-7 and 83-14 was similar to that with insulin (Fig. 2g), while D707A was activated by antibodies but not insulin (Fig. 2j).

We next evaluated responses to insulin +10 nmol/l antibody, based on evidence that this concentration elicits the maximal response [18, 20]. In the presence of antibodies 83-7 and 83-14, the maximal response of WT and mutant INSRs to insulin was increased without affecting potency (although EC_50_ values were not precisely determined) (Fig. 2k–t, Table 2). This was observed across all mutant receptors except the kinase-dead P1178L [24, 25]. Antibodies 18-44 and 18-146 elicited smaller effects than 83-7 and 83-14, and for clarity of presentation data for these antibodies are shown in the ESM (ESM Results, ESM Figs 1, 2, ESM Table 7).

### Generation of a novel adipocyte cell model of insulin receptoropathy

**Fig. 3:**
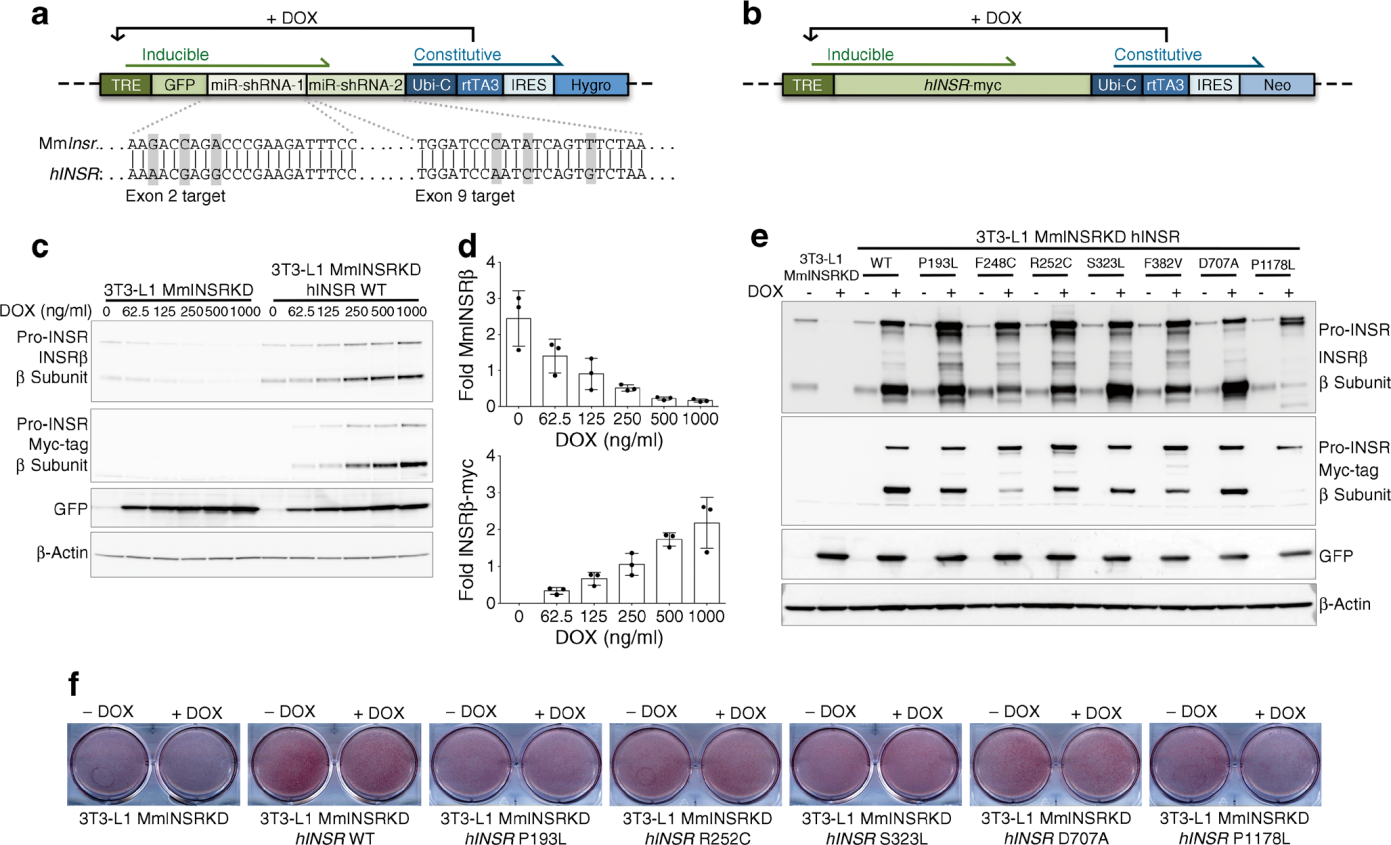
Generation of a novel stable 3T3-L1 adipocyte model of insulin receptoropathy. (**a**) Concatenated miR-shRNAs targeting murine *Insr* in exon 2 and exon 9 preceded by GFP under the control of a tet-responsive element was packaged into third-generation lentivirus to enable transduction of 3T3-L1 pre-adipocytes. The exploded view shows the nucleotide mismatches between the mouse *Insr* targeted by each miR-shRNA with the human *INSR* sequence. Green shaded elements of the transgene are inducible by the addition of DOX. Transduced 3T3-L1 pre-adipocytes underwent single cell clonal selection in the presence of hygromycin to generate 3T3-L1 MmINSRKD. (**b**) 3T3-L1 MmINSRKD cells were then transduced with a second lentivirus encoding C-terminal myc-tagged human *INSR* transgenes under the control of a tet-responsive element and underwent polyclonal selection in the presence of neomycin to generate 3T3-L1 MmINSRKD *hINSR*. (**c**) Western blots of whole-cell lysates from day 10 mature 3T3-L1 MmINSRKD and 3T3-L1 MmINSRKD *hINSR* WT cells grown in the presence of increasing concentrations of DOX for 72 h. (**d**) Densitometry analysis of western blots from three independent experiments demonstrating knockdown of endogenous mouse *Insr* and expression of human *INSR* with increasing concentrations of DOX. (**e**) Western blots of whole-cell lysates from day 16 mature 3T3-L1 MmINSRKD and 3T3-L1 MmINSRKD *hINSR* (mutant INSR as indicated) cells grown in the presence of 1 μg/ml DOX for 10 days. (**f**) Oil Red O staining of lipid accumulation in day 10 mature 3T3-L1 MmINSRKD and 3T3-L1 MmINSRKD *hINSR* WT or mutant (as indicated) cells grown ± DOX (1 μg/ml) for 72 h. GFP, green fluorescent protein; Hygro, hygromycin resistance; IRES, internal ribosome entry site; Mm, murine; Neo, neomycin resistance; rtTA3, reverse tetracycline-controlled transactivator; TRE, tet-response element; Ubi-C, ubiquitin C promoter

To assess antibody-induced signalling downstream from the INSR, an adipocyte model of insulin receptoropathy was generated. A tetracycline (tet)-responsive microRNA (miR)-short hairpin (sh)RNA selectively targeting murine *Insr* was transduced into 3T3-L1 pre-adipocytes to generate a stable clone (Fig. 3a). This was transduced with lentiviruses encoding C-terminal myc-tagged WT or mutant *hINSR*, also controlled by tet-responsive elements (Fig. 3b), generating cells in which DOX simultaneously knocked down endogenous murine *Insr* and induced overexpression of myc-tagged human INSR. This system permitted pre-adipocyte differentiation uncompromised by mutant receptor expression before induction of *Insr* knockdown/*hINSR* re-expression in adipocytes (Fig. 3c, d). The DOX concentration producing maximal *Insr* knockdown resulted in overexpression of *hINSR* transgenes (Fig. 3c, e); however, the receptor-processing defects observed in CHO cells (Fig. 1c) were preserved. The C-terminal myc-tag enabled discrimination of endogenous mouse and ectopic human INSR by size shift of the INSR β subunit on immunoblotting, or by anti-myc antibodies (Fig. 3e). The pre-adipocyte cell lines generated differentiated efficiently into mature adipocytes, as evidenced by Oil Red O staining (Fig. 3f).

### Activation of signalling downstream from mutant INSRs by insulin and antibody

**Fig. 4:**
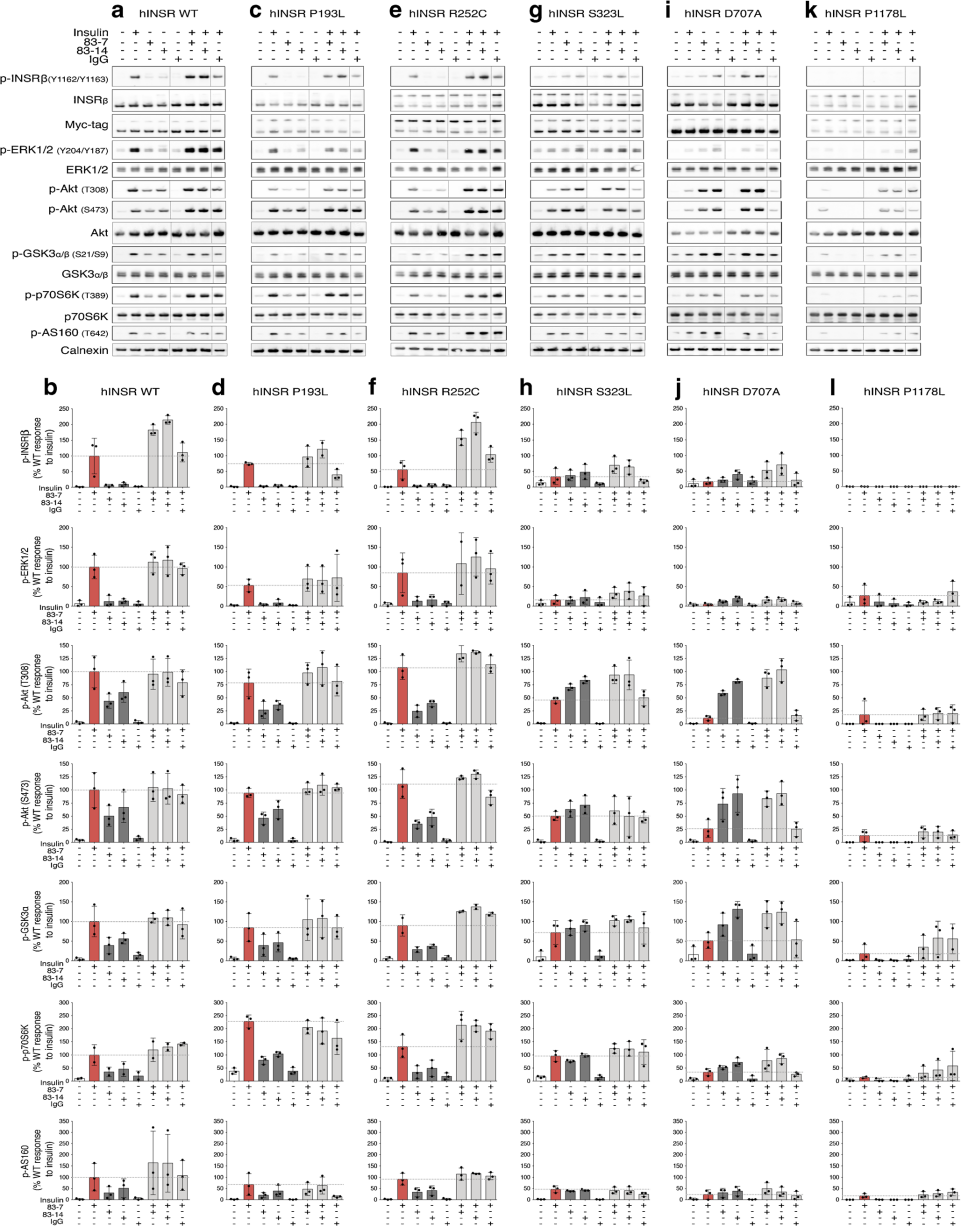
Activation of signalling pathways downstream of WT and mutant INSR by insulin and antibody stimulation. 3T3-L1 MmINSRKD *hINSR* WT (**a**, **b**), P193L (**c**, **d**), R252C (**e**, **f**), S323L (**g**, **h**), D707A (**i**, **j**) and P1178L (**k**, **l**) adipocytes were grown in the presence of 1 μg/ml DOX for 8 days prior to overnight serum starvation on day 13 of differentiation. Adipocytes were then stimulated with either 10 nmol/l insulin (red bars), 10 nmol/l antibody (83-7, 83-14 or control IgG; dark grey bars) or 10 nmol/l insulin containing 10 nmol/l antibody (light grey bars) for 10 min at 37°C/5% CO_2_. Following stimulation, cells were washed and snap frozen prior to lysis and western blot. Bar graphs show p-INSRβ, p-ERK1/2, p-Akt, p-GSK3α, p-p70S6K and p-AS160 densitometry after normalisation for each sample by the sum aggregate of multiple proteins (total INSRβ, myc-tagged INSRβ, ERK1/2, Akt, GSK3α/β, p70S6K and calnexin) for each biological replicate. Data are the mean ± SD of three independent experiments and are expressed relative to *hINSR* WT response to insulin stimulation; individual data points are shown in scatter plots. For clarity of presentation, only key data are presented here; an extended version appears as ESM Fig. 2

Plasma insulin concentration in human insulin receptoropathies lies between 0.3 and 3 nmol/l in the fasting state (ESM Table 6), and at least an order of magnitude higher when fed. We used an insulin concentration of 10 nmol/l, mimicking the fed disease state. WT INSR autophosphorylation was strongly induced by insulin (Fig. 4a, b), but was undetectable after receptor knockdown alone (ESM Fig. 2c, d). Otherwise, the pattern of autophosphorylation of overexpressed receptors in response to insulin and/or antibody was similar to that seen in CHO cells. Thus, antibodies 83-7 and 83-14 alone induced WT receptor autophosphorylation on Y1162/Y1163, while antibodies 18-44 and 18-146 were less effective (ESM Fig. 2, ESM Results). Insulin-stimulated autophosphorylation was reduced by 75-100% in mutant INSRs compared with WT. Although antibodies alone induced low-level phosphorylation of mutant INSRs (<10%), the responses of S323L and D707A to antibodies 83-7 and 83-14 were equal to or greater than those to insulin (Fig. 4g–j). Combined insulin and antibody treatment enhanced phosphorylation of each mutant INSR in the case of 83-7 and 83-14, likely synergistically (Fig. 4, ESM Fig. 2).

Akt2/PKBβ transduces metabolic actions of insulin after phosphorylation of T308 and S473. p-Akt2 phosphorylates substrates including glycogen synthase kinase (GSK3α/β), which regulates glycogen synthesis, p70 S6 kinase (p70S6K), which stimulates protein synthesis, and AS160, which encodes a GTPase-activating protein that restrains GLUT4 vesicle translocation until phosphorylated. Insulin treatment of WT INSR induced strong Akt phosphorylation at both sites (Fig. 4a, b), and this was severely attenuated by knockdown of endogenous *Insr* or by knockdown with re-expression of the kinase-dead P1178L mutant (Fig. 4k, l). Attenuation of signalling was also apparent downstream of Akt, with phosphorylation of p70S6K and GSK3 only modestly impaired, and AS160 phosphorylation unaffected.

Several patterns were seen across the panel of mutants studied. In D707A receptor-expressing cells (Fig. 4i, j), insulin-induced phosphorylation of Akt and its substrates was severely attenuated, while a progressive ‘escape’ from signalling impairment was seen in mutants S323L (Fig. 4g, h), F248C (ESM Fig. 2g, h) and F382V (ESM Fig. 2m, n), with lesser impairment of Akt phosphorylation than of receptor autophosphorylation, and only partial inhibition at downstream substrates. P193L (Fig. 4c, d) and R252C (Fig. 4e, f) demonstrated similar insulin-induced Akt and Akt substrate phosphorylation to WT receptor.

Antibodies alone stimulated Akt and Akt substrate phosphorylation in all cells except those overexpressing the kinase-dead P1178L mutant (Fig. 4k, l). For S323L and D707A mutants, antibodies 83-7 and 83-14 stimulated greater phosphorylation than insulin alone, by virtue of the low response of those mutants to insulin (Fig. 4g–j). Co-treatment of cells with insulin and antibodies 83-7 and 83-14 enhanced Akt and Akt substrate phosphorylation with respect to insulin alone, without evidence of synergy. The additive effects of insulin and antibody co-stimulation were generally less than those observed for receptor autophosphorylation in CHO cells (Fig. 2k–t).

Activation of the INSR by insulin stimulates not only phosphoinositide 3-kinase (PI3K)/Akt, but also RAS/RAF/mitogen-activated protein kinase kinase (MEK)/ERK signalling, through both IRS-dependent and IRS-independent mechanisms [31]. Activation of this pathway is a surrogate for mitogenicity of insulin analogues [32], which is important in view of concerns about long-term cancer risks of analogues with pro-proliferative activity. Insulin treatment of WT INSR induced robust phosphorylation of ERK1/2 at Y204/Y187, with each mutant INSR displaying reduced phosphorylation in response to insulin compared with WT (Fig. 4). Antibody treatment of mutant or WT INSR did not induce ERK1/2 phosphorylation, while dual stimulation with antibody + insulin did not increase ERK1/2 phosphorylation compared with insulin alone. Higher basal ERK1/2 phosphorylation was observed in cells with *Insr* knockdown alone (ESM Fig. 2c, d), or with *Insr* knockdown and P1178L receptor overexpression (Fig. 4k, l), but this did not change with any treatment.

### Effect of insulin and/or antibody on glucose uptake

**Fig. 5:**
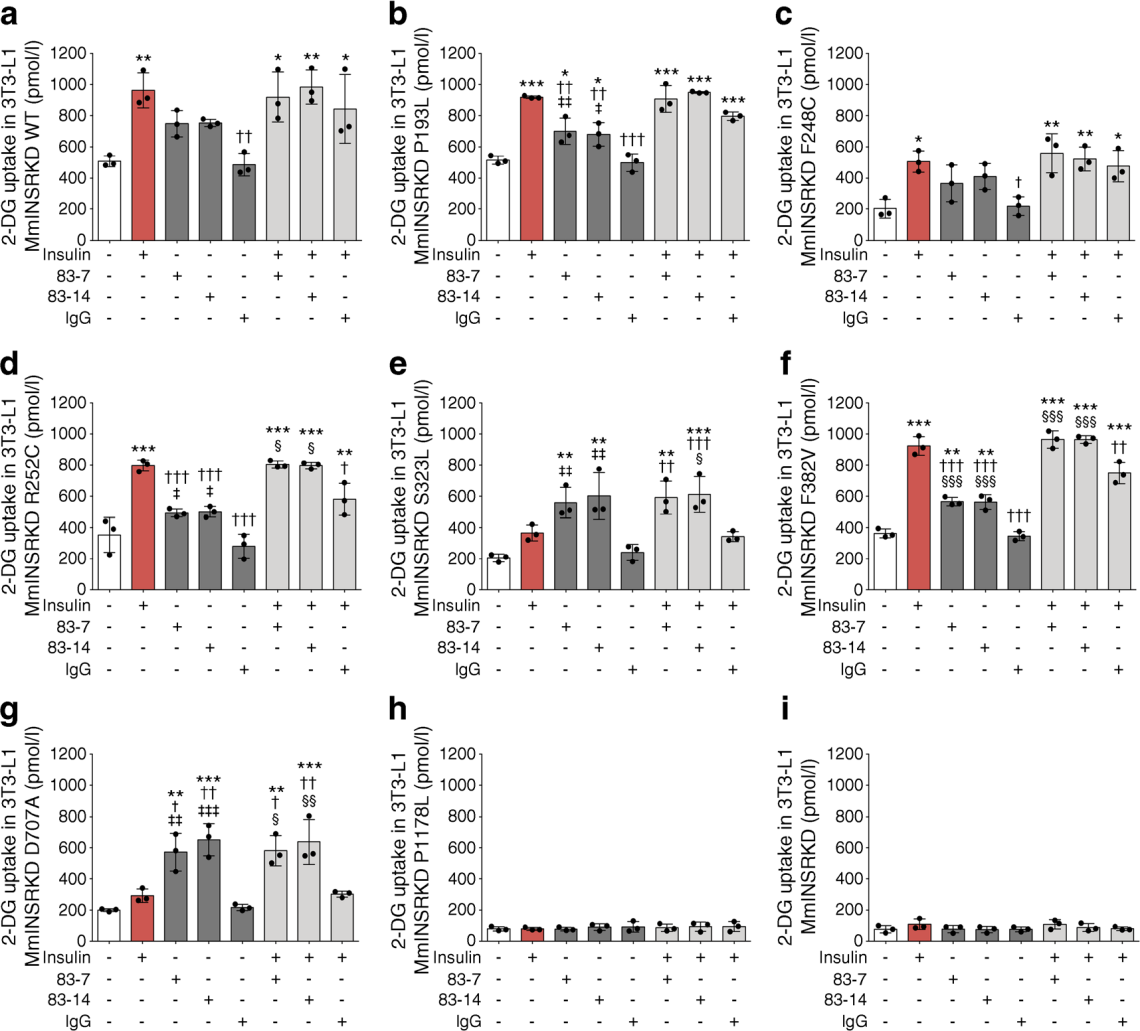
Insulin- and antibody-stimulated glucose uptake via WT and mutant INSR. 3T3-L1 MmINSRKD *hINSR* WT (**a**), P193L (**b**), F248C (**c**), R252C (**d**), S323L (**e**), F382V (**f**), D707A (**g**), P1178L (**h**) and MmINSRKD (**i**) adipocytes were grown in the presence of 1 μg/ml DOX for 10 days prior to overnight serum starvation on day 15 of differentiation. The cells were stimulated for 30 min with either 10 nmol/l insulin (red bars), 10 nmol/l antibody (83-7, 83-14 or control IgG; dark grey bars) or 10 nmol/l insulin containing 10 nmol/l antibody (light grey bars) prior to the addition of 2-deoxy-d-glucose for 5 min. Cells were then washed, lysed and assessed for 2-deoxy-d-glucose uptake. Bar chart data are the mean ± SD from three independent experiments; scatter plots indicate the mean of triplicates from each independent experiment. Statistical significance was determined by one-way ANOVA with Tukey’s multiple comparison test: ^*^p < 0.05, ^**^p < 0.01 and ^***^p < 0.001, vs unstimulated basal; ^†^*p* < 0.05, ^††^*p* < 0.01 and ^†††^*p* < 0.001, vs 10 nmol/l insulin treatment; ^‡^*p* < 0.05, ^‡‡^*p* < 0.01 and ^‡‡‡^*p* < 0.001, vs 10 nmol/l IgG control treatment; ^§^*p* < 0.05, ^§§^*p* < 0.01 and ^§§§^*p* < 0.001, vs 10 nmol/l insulin in the presence of 10 nmol/l IgG control. 2-DG, 2-deoxy-d-glucose

Glucose uptake is a key outcome of INSR activation and was assessed in the 3T3-L1 model. Parent 3T3-L1 cells and cells harbouring the *Insr*-knockdown construct but not treated with DOX displayed similar high levels of insulin-stimulated glucose uptake (ESM Fig. 3a, b), but insulin did not stimulate uptake in conditional *Insr*-knockdown cells treated with doxycycline (Fig. 5i). Cells with endogenous mouse *Insr* knockdown and WT *hINSR* re-expression, in contrast, demonstrated only a 1.8-fold increase in glucose uptake on insulin stimulation (ESM Fig. 3a). The apparently poor response to insulin was due to increased basal glucose uptake in WT receptor-overexpressing cells (ESM Fig. 3b). Basal uptake among mutant receptor-expressing cell lines reflected mutant receptor function (ESM Fig. 3c).

Despite reduced dynamic range in the assay, insulin stimulated glucose uptake via WT, P193L, F248C, R252C and F382V receptors (Fig. 5a, b, d, f, respectively). Insulin-stimulated uptake was similar in cells expressing P193L, R252C or F382V receptor and those expressing WT receptor, but was reduced in cells expressing the F248C mutant. No stimulation of glucose uptake was seen in cells expressing S323L, D707A or P1178L receptors (Fig. 5e, g, h, respectively).

Antibodies 83-7 and 83-14 alone stimulated glucose uptake via P193L, S323L, F382V and D707A receptors (Fig. 5), while antibodies 18-44 and 18-146 were again less effective across the full range of mutants (ESM Fig. 4). While the magnitude of antibody-stimulated uptake was less than that seen with insulin via WT, P193L, F248C, R252C and F382V receptors, antibodies 83-7, 83-14 and 18-44 were more effective than insulin at stimulating glucose uptake via D707A. Dual treatment with antibodies + insulin did not enhance glucose uptake compared with insulin alone acting via WT, P193L, F248C, R252C and F382V receptors, or antibody alone when acting via S323L and D707A receptors.

## Discussion

Recessive insulin receptoropathies feature failure to thrive, extreme metabolic derangement, childhood mortality and poor response to therapy. Longitudinal studies suggest a steep relationship between residual INSR function and clinical outcome: loss of 50% INSR function, as in the parents of infants with Donohue syndrome, does not produce insulin resistance in lean people. Heterozygous dominant negative mutations produce severe insulin resistance, diagnosed peripubertally in girls and later in men, and reduce receptor function to 25% or less of WT. The severe recessive receptoropathies that this study focuses on confer greater loss of function. However, even with 0–25% residual function, a range of phenotypes is seen, with complete loss of function producing Donohue syndrome and lethality in infancy, but less extreme loss of function producing Rabson–Mendenhall syndrome, with survival to the second or third decade. These observations suggest that even modest improvements in receptor signalling in recessive disease may have decisive clinical benefit.

Many pathogenic *INSR* mutations are known, including more than 100 missense mutations. A subset are expressed at the cell surface, but show impaired insulin binding, signal transduction or internalisation and recycling. This subset may be amenable to non-conventional activation by antibody. Proof of this principle came from demonstration that two bivalent antibodies stimulated kinase activity of a single solubilised mutant receptor (F382V [7]), and, independently, that one bivalent antibody increased glycogen synthesis acting via a mutant receptor expressed in intact cells (S232L [8]). We extend these findings with systematic characterisation of multiple receptor mutants and antibodies in two cellular systems, assaying physiologically important responses including adipocyte glucose uptake.

One of the mutants assessed, F248C, is novel. It lies close to the R252C mutant, which is expressed but exhibits impaired internalisation after insulin exposure [33]. F248C shows minor reduction in cell surface expression, but insulin-stimulated receptor autophosphorylation and downstream signalling are severely impaired. Across known mutants, our data generally agree with prior studies. Assay of receptor autophosphorylation in CHO cells using immunocapture of myc-tagged receptor prior to immunoassay demonstrated signalling defects more clearly than phosphotyrosine immunoblotting in the 3T3-L1 overexpression system, likely reflecting the inherently greater dynamic range of immunoassay allied to use of a generic anti-phosphotyrosine antibody.

We confirmed that S323L and F382V receptors can be activated by antibodies and extended these observations to a wider range of mutants. Previous studies suggest that receptor activation by antibody depends on receptor cross-linking rather than reaction at specific epitopes [19]. Consistent with this, two of the antibodies we employed, 83-7 and 83-14, are both effective despite recognising different epitopes and having different effects on insulin binding. Antibodies 18-44 and 18-146 consistently elicited much smaller responses, although 18-44 has previously been found to exert insulin-like activity on primary human adipocytes [20]. Differences among antibodies are likely to reflect differences of affinity and/or steric constraints on cross-linking receptors.

The mutants showing the largest antibody response were S323L and D707A, both being activated by antibodies similarly to WT receptor, and to a greater extent than by insulin. Such mutants with ‘pure’ insulin-binding defects are particularly attractive therapeutic targets. Other mutants studied in both cell systems (P193L, F248C, R252C and F382V) showed some activation of Akt, GSK3, AS160 and glucose uptake by antibodies. In these cases, responses were less than for WT receptor or those induced by insulin. Testing the therapeutic potential of antibodies against such mutants is warranted in vivo, where antibody signalling may be prolonged compared with insulin signalling because of slower receptor internalisation. Indeed, a previously studied anti-INSR antibody showed markedly greater hypoglycaemic effects in vivo in WT animals than had been apparent in cell culture models [13].

Antibodies would be a particularly appealing therapeutic proposition were they to exhibit synergy with insulin in receptor stimulation, amplifying insulin action rather than simply imposing a tonic signal. The current studies have not addressed this in detail, although suggestive evidence for synergic stimulation of WT receptor and some mutant receptors is seen. This was not mirrored by detectable synergistic activation of downstream signalling or metabolic endpoints, possibly because maximal downstream signalling requires only submaximal receptor autophosphorylation. It remains possible that insulin–antibody synergy does exist but was obscured under the conditions of the experiments undertaken, which pragmatically employed relatively high concentrations of insulin and antibody.

Early cellular studies of antibody-induced INSR activation were interpreted as suggesting that antibodies elicit greater downstream responses than expected from low levels of receptor autophosphorylation [16, 34–36]. These observations were later argued to have a methodological basis, hinging on lower sensitivity in detecting tyrosine phosphorylation than downstream signalling [37, 38]. This is, in part, because signal amplification is an inherent property of signal transduction cascades. Our observation of apparent ‘escape’ from signalling inhibition in the face of efficient *Insr* knockdown in 3T3-L1 adipocytes supports this contention, as activation of residual receptors is undetectable directly but is observable downstream, owing to signal amplification.

Importantly, receptor activation by antibodies leads to selective Akt phosphorylation, which is critical for metabolic actions of insulin, with little or no ERK phosphorylation. As activation of the RAS/RAF/MEK/ERK pathway is mitogenic, this is an encouraging property of antibodies for translational purposes, suggesting that they may exert metabolic benefits without undue mitogenic activity. Similar dissociation between activation of Akt and ERK has also been observed following INSR activation by the peptide ligand S597 [39] and in previous studies with anti-receptor antibodies [40]. The mechanism underlying such biased agonism is poorly understood, although IRS proteins may be preferentially phosphorylated by plasma membrane-associated receptor [33, 41], whereas receptor internalisation is required for full ERK activation [33, 42].

We studied only a limited number of insulin and antibody concentrations. While these were selected with reference to prior studies and observed blood insulin concentrations in insulin receptoropathy, the conditions we describe may not be most relevant in vivo, where insulin and antibody concentrations in the interstitial space of target tissues may be variable and different. Moreover, receptor overexpression may have partially overcome receptor dysfunction and made beneficial effects of antibody more difficult to observe. Finally, in the paradigm of acute antibody stimulation with static signalling endpoints, issues such as the potential of long-term antibody treatment to downregulate receptors, and the effect of antibodies on receptor recycling kinetics in vivo have not been addressed. This is likely to be particularly important for the subset of mutants (e.g. I119M, K460E) where acute insulin stimulations studies are normal, as in this and other reports, but which confer extreme insulin resistance in vivo.

## Conclusions

Multiple monoclonal antibodies can bind and activate mutated cell surface INSR to a potentially clinically significant degree. Experience in WT animals [13] and theoretical considerations argue that effects of anti-INSR antibodies in vivo may be greater than in cells, so further studies in animal models are warranted.

## Electronic supplementary material

ESM (PDF 9825 kb)

## Data Availability

All data generated or analysed during this study are included in this published article and the ESM.

## Abbreviations

3T3-L1 MmINSRKD: Murine *Insr*-knockdown cells
AS160: Akt substrate of 160 kDa
CHO: Chinese hamster ovary
DOX: Doxycycline
ERK1/2: Extracellular signal-regulated kinase 1/2
GSK3: Glycogen synthesis kinase 3
INSR: Insulin receptor
MEK: Mitogen-activated protein kinase kinase
MOI: Multiplicity of infection
p70S6K: Ribosomal protein S6 kinase β-1
shRNA: Short hairpin RNA
tet: Tetracycline
TIFF: Tag image file format
WT: wild-type
