# Supplementary material for: Evaluation of anti-insulin receptor antibodies as potential novel therapies for human insulin receptoropathy using cell culture models
Source: Diabetologia. 2018 Apr 27;61(7):1662–75. doi: 10.1007/s00125-018-4606-2 (PMC6445487; doi:10.1007/s00125-018-4606-2)
Supplement: Supplementary file 1 — (PDF 9825 kb) [file 125_2018_4606_MOESM1_ESM.pdf]

Brierley *et al.* ESM Content

ESM Results ______________ **page 2**

ESM Table 1 ______________ **page 4**

ESM Table 2 ______________ **page 4**

ESM Table 3 ______________ **page 5**

ESM Table 4 ______________ **page 6**

ESM Table 5 ______________ **page 6**

ESM Table 6 ______________ **page 7**

ESM Table 7 ______________ **page 8**

References for ESM Table 7 __ **page 10**

ESM Fig. 1 ______________ **page 12**

ESM Fig. 2 ______________ **page 14**

ESM Fig. 3 ______________ **page 16**

ESM Fig. 4 ______________ **page 17**

1. ESM Results

Antibodies 18-44 and 18-146 elicited more modest effects than 83-7 and 83-14, therefore for clarity of presentation, results pertaining to experiments conducted with antibodies 18-44 and 18-146 are discussed here.

1.1 Assessment of mutant INSR autophosphorylation in response to antibody and/or insulin

Antibody 18-146 alone was able to elicit autophosphorylation of R118C and K460E (ESM Figure 1), however the magnitude of this response was substantially lower than that of the corresponding insulin response (ESM Table 7). Antibody 18-44 alone, stimulated I119M autophosphorylation, but again this was substantially lower than that of the response elicited by insulin (ESM Fig.1, ESM Table 7). In the presence of 10nM antibody the maximum efficacy of insulin in activating both WT and mutant INSRs was increased (ESM Fig. 1 B, D, F, H, L, N, P, R, T). Similar to that observed with antibodies 83-7 and 83-14, the increase in maximal efficacy was not accompanied by a change in potency (ESM Table 7). However, EC_{50} values were not determined with precision due to the small magnitude of responses and limited data points. The data for mutants S323L and D707A suggest that an extended treatment range would reveal an effect of antibody treatment on insulin potency (ESM Fig. 1 N & T).

1.2 Activation of signaling downstream from mutant INSRs by insulin and antibody

ESM Figure 2 displays the full extent of the signaling studies undertaken. A similar pattern of mutant INSR autophosphorylation stimulated by antibodies 18-44 or 18-146 either alone or in combination with insulin was observed in the 3T3-L1 insulin receptoropathy model (ESM Fig. 2) as observed in the lysates from CHO cells (ESM Fig. 1). Thus, antibodies 18-44 and 18-146 alone did not appreciably activate WT or mutant INSR. However, the increase in mutant INSR autophosphorylation upon stimulation with insulin in the presence of antibody was not observed (ESM Fig. 2), possibly reflecting the difference in sensitivity between the two assays (immunoassay vs. Western blot). Antibody 18-44 and 18-146 stimulation of WT (ESM Fig. 2A, B), P193L (ESM Fig. 2E, F), S323L (ESM Fig. 2K, L) and D707A (ESM Fig. 2O, P) lead to very modest phosphorylation

of Akt and its downstream substrates. However, antibody 18-44 and 18-146 stimulation of WT and mutant INSR did not result in appreciable ERK phosphorylation, nor did they potentiate insulin stimulated ERK phosphorylation. This broadly reflects the selective stimulation of Akt over ERK phosphorylation observed with antibodies 83-7 and 83-14.

1.3 Effect of insulin and/or antibody on glucose uptake

Insulin significantly stimulated glucose uptake via WT, P193L, F248C, R252C, and F382V INSR but did not have a significant effect acting via S323L or D707A mutant receptors (ESM Fig. 4). Only treatment with antibody 18-44 alone significantly stimulated glucose uptake via one mutant, D707A (ESM Fig.4G). Dual treatment of insulin in the presence of antibody did not have any effect on the ability of insulin to stimulate glucose uptake via WT, P193L, F248C, R252C, and F382V INSR. However, stimulation of S323L by insulin in the presence of 18-146 did significantly stimulate glucose uptake over basal ($p<0.001$), but was not statistically significant from that of insulin treatment alone (ESM Fig. 4E). Likewise, stimulation of D707A with insulin in the presence of either 18-44 or 18-146 significantly increased glucose uptake over that of basal conditions ($p<0.01$ and $p<0.05$, respectively), but was not statistically significant from that of insulin treatment alone (ESM Fig. 4G).

ESM Table 1: Composition of cell culture medium

Cell Line	Media Name	Base Medium	Supplements
CHO FlpIN	CHO Media	F12	10% (v/v) foetal calf serum (FCS), 50 units/ml penicillin, 50units/ml streptomycin, 4mM L-glutamine
3T3-L1 Preadipocytes	Preadipocyte Media	DMEM	10% (v/v) newborn calf serum (NCS), 50 units/ml penicillin, 50units/ml streptomycin, 4mM L-glutamine
3T3-L1 Adipocytes	Adipocyte Media	DMEM	10% (v/v) TET-approved FCS (Clontech), 50 units/ml penicillin, 50units/ml streptomycin, 4mM L-glutamine
3T3-L1 Preadipocytes	Differentiation media 1	DMEM	Same as Adipocyte media with the addition of 1μM insulin, 200nM rosiglitazone, 500μM methylisobutylxanthine, 1μM dexamethasone
3T3-L1 Adipocytes	Differentiation media 2	DMEM	Same as Adipocyte media with the addition of 1μM insulin and 200nM rosiglitazone

ESM Table 2: Vectors and sub-cloning steps used in generation of CHO FlpIn hINSR cells

Vector	Source/Reference	Use
pCR_Blunt_II_TOPO	Invitrogen	TOPO clone hINSR PCR product from pDNR-Dual
pCDNA/5/FRT/TO	Invitrogen	hINSR expression vector<br>ApaI/HindIII hINSR fragment from pCR_Blunt_II_TOPO
pOG44	Invitrogen	Expression of Flp recombinase

ESM Table 3: Target sequences, primers, vectors and sub-cloning steps used in the generation of lentiviruses

Sequence/vector	Source/Reference	Use
CGGATCCCATATCAGTTTCTAA	Open Biosystems	Target sequence for murine INSR miR-shRNA
AAGACCAGACCCGAAGATTTCC	Seibler *et al (2007)* *Nucleic Acids Res.* **35**, e54	Target sequence for murine INSR miR-shRNA
pEN-TGmiRC3	Shin *et al (2006)* PNAS **103**, 13759–13764 (2006)	miR-shRNAs concatenated into this entry vector by SpeI, XbaI, PstI directional cloning as described by Shin *et al*
pSLIK-Hygro	Shin *et al (2006)* PNAS **103**, 13759–13764 (2006)	miR-shRNAs gateway cloned into this lentiviral expression vector by gateway cloning with LR clonase
pMDLg/pRRE, pRSVREV, pVSV-G	Shin *et al (2006)* PNAS **103**, 13759–13764 (2006)	Third-generation lentivirus packaging and pseudotyping plasmids
pEN_Tmcs	Shin *et al (2006)* PNAS **103**, 13759–13764 (2006)	Entry vector
pEN_TmcsMCS2	N/A	Oligonucleotide linker encoding NotI-BamHI-ScaI-SphI-HindIII-NcoI-PmeI-KpnI-ApaI-XhoI was cloned into pEN_Tmcs between NotI and XhoI sites. PCR amplified hINSR cloned into SpeI and HindIII sites.
GGGGACTACTTCCACCATGGCCACCG	Sigma-Aldrich	Fwd 5’-3’ primer to amplify myc-tagged hINSR mutants from pCDNA5/FRT/TO
GCATGCAAGCTTCTACAGATCCTCTTC TGAGATGAG	Sigma-Aldrich	Rev 5’-3’ primer to amplify myc-tagged hINSR mutants from pCDNA5/FRT/TO
pSLIK-NEO	Shin *et al (2006)* PNAS **103**, 13759–13764 (2006)	Mutant hINSR cloned into this expression vector by gateway cloning from pEN_TmcsMCS2

ESM Table 4: Buffer Composition

Buffer Name	Composition
FACS Buffer	PBS, 0.5% BSA, 0.1% sodium azide
Lysis Buffer	20mM HEPES, 150mM NaCl, 1.2mM $MgCl_2$, 1mM EGTA, 1mM PMSF, 1mM Na_3VO_4, 10% (v/v) glycerol, 1% (v/v) Triton-X-100, complete-EDTA-free protease inhibitors (Roche), phosSTOP (Roche)
KRPH Buffer	120mM NaCl, 5mM KCl, 1.2mM $MgCl_2$, 10mM $NaHCO_3$, 1.3mM $CaCl_2$, 1.2mM KH_2PO_4, 20mM HEPES

ESM Table 5: Antibodies used during Western blotting

Target	Dilution	Catalogue #	Manufacturer
INSRβ	1:200	SC-711	Santa Cruz Biotechnology
INSRβ	1:1000	3025	Cell Signalling Technology
Phospho-INSRβ (Tyr1162/Tyr1163)	1:1000	44804G	Life Technologies
Myc-tag	1:1000	05-724	Millipore
Calnexin	1:1000	Ab22595	Abcam
Phospho-Akt (Thr308)	1:1000	2965	Cell Signalling Technology
Phospho-Akt (Ser473)	1:1000	4060	Cell Signalling Technology
Akt	1:1000	2920	Cell Signalling Technology
Phospho-ERK1/2 (Tyr204/Tyr187)	1:1000	5726	Cell Signalling Technology
ERK1/2	1:1000	4695	Cell Signalling Technology
Phospho-GSK3α/β (Ser21/Ser9)	1:1000	9331	Cell Signalling Technology
GSK3α/β	1:1000	5676	Cell Signalling Technology
Phospho-p70S6K (Thr389)	1:1000	9205	Cell Signalling Technology
p70S6K	1:1000	2708	Cell Signalling Technology
Phospho-AS160	1:1000	4288	Cell Signalling Technology
Anti-mouse IgG HRP conjugated	1:10,000	7076	Cell Signalling Technology
Anti-rabbit IgG HRP-conjugated	1:5,000	7074	Cell Signalling Technology

ESM Table 6. Characteristics of mutant INSR and patient phenotypes

Mutation	INSR subunit	Phenotype	Plasma insulin pmol/L	INSR defect characteristics				Cell types used to characterise mutant
				Cell surface expression	Insulin binding	Insulin-stimulated autophosphorylation	Internalisation, dissociation, degradation	
L62P	α	TA-IR	600 – 3000*(1)	✖	↓(1)	↓(1)	NA	RBC(1), HEK293(2)
R118C	α	TA-IR, RMS	70 - 3000(3)	N(3)	↓(3)	↓(3)	NA	CHO(3)
I119M	α	TA-IR, RMS	2000 – 20000*(4)	N(4)	N(4)	N(4)	Dissociation ↓(4)	EBVL(4), CHO(4)
P193L	α	RMS	1000 – 2000*(5)	↓(6)	↓(5)	NA	NA	EBVL(5), Rat-1(6)
F248C	α	DS	7000	◆	◆	◆	◆	◆
R252C	α	TA-IR	NR	N(7)	↓(7)	N(7)	Internalisation ↓(7)	CHO(7)
S323L	α	DS, RMS	2000 – 8000(8,9)	N(8, 10)	↓(8, 10, 11)	↓(8, 10, 11)	NA	PBMC(8), NIH-3T3(8, 10), CHO(11)
F382V	α	TA-IR	NR	↓(12)	N(12, 13)	↓(13)	NA	NIH-3T3(12, 13)
K460E	α	DS, RMS	1000 – 70000(14, 15)	N(15)	↑(16)	N/↑(14)	Int. ↑(14), diss.↓ (17), deg. ↑(14)	EBVL(16, 17), PBMC(15), PDF(15), NIH-3T3(14)
D707A	α	DS	2000 – 3000*(18)	N(18)	↓(18)	✖(18)	Internalisation ↓(18)	PDF(18), CHO(18)
P1178L	β	TA-IR	2000 - >4000(9)	N(19, 20)	N(19, 20)	✖(19, 20)	NA	CHO(19, 20)

Previously published characteristics of naturally occurring INSR mutations used in the current study. Mutations are numbered as per the mature INSR B isoform (exon 11+). TA-IR, Type-A Insulin Resistance; RMS, Rabson Mendenhall Syndrome; DS, Donohue Syndrome; *, reported fasting insulin levels; NR, not reported; ✖, absent; N, normal; ↓, decreased compared to WT receptor; ↑, increased compared to WT receptor; ◆, not previously described; NA, not assessed; RBC, red blood cells; EBVL, Epstein Barr virus transformed lymphoblasts; PBMC, peripheral blood mononucleocytes; CHO, Chinese Hamster Ovary; Rat-1, Rat-1 fibroblasts; NIH-3T3, NIH-3T3 murine fibroblasts; HEK293, human embryonic kidney 293.

ESM Table 7.

Autophosphorylation of WT and mutant INSR stimulated by insulin, antibodies, or insulin plus 10nM antibody (18-44 & 18-146)

EC_{50}, half-maximal effective concentration in nmol/l; pEC_{50}, negative log of EC_{50} value in mol/l; 95% CI pEC_{50}, 95% confidence interval of pEC_{50}; E_{max}, maximum efficacy expressed as a percentage of a particular receptors response to insulin (% Ins) or as a percentage of wild type receptor response to insulin (% WT Ins); −, not able to be determined. Graphical data is presented in ESM Figure 1 on page 10.

Treatment	WT				R118C					I119M				
	EC_{50}	pEC_{50}	95% CI pEC_{50}	E_{max} (% Ins)	EC_{50}	pEC_{50}	95% CI pEC_{50}	E_{max} (% Ins)	E_{max} (% WT Ins)	EC_{50}	pEC_{50}	95% CI pEC_{50}	E_{max} (% Ins)	E_{max} (% WT Ins)
Insulin	0.3	9.5	(9.7 – 9.4)	100	1.5	8.8	(9.1 - 8.5)	100	65	2.6	8.6	(8.9 – 8.3)	100	99
18-44	−	−	−	1	−	−	−	4	3	−	−	−	11	11
18-146	−	−	−	4	−	−	−	31	20	−	−	−	3	3
IgG	−	−	−	0	−	−	−	3	2	−	−	−	1	1
Insulin + 18-44	2.5	8.6	(8.9 - 8.3)	83	1.9	8.7	(9.0 - 8.4)	112	73	3.4	8.5	(8.6 – 8.3)	107	106
Insulin + 18-146	2.9	8.5	(8.8 - 8.2)	105	1.7	8.8	(9.0 - 8.5)	148	97	3.3	8.3	(8.6 - 8.3)	125	125
Insulin + IgG	3.4	8.5	(9.0 – 7.9)	64	1.4	8.8	(9.1 - 8.6)	94	61	3.4	8.5	(8.6 – 8.3)	93	92

ESM Table 7 Continued…

Treatment	P193L					F248C					R252C				
	EC_{50}	pEC_{50}	95% CI pEC_{50}	E_{max} (% Ins)	E_{max} (% WT Ins)	EC_{50}	pEC_{50}	95% CI pEC_{50}	E_{max} (% Ins)	E_{max} (% WT Ins)	EC_{50}	pEC_{50}	95% CI pEC_{50}	E_{max} (% Ins)	E_{max} (% WT Ins)
Insulin	1.4	8.9	(9.1 - 8.7)	100	23	0.4	7.2	(8.4 - 6.1)	100	3	2.2	8.6	(9.0 - 8.3)	100	27
18-44	−	−	−	9	2	−	−	−	0	0	−	−	−	4	1
18-146	−	−	−	4	1	−	−	−	0	0	−	−	−	4	1
IgG	−	−	−	0	0	−	−	−	0	0	−	−	−	0	0
Insulin + 18-44	0.8	9.1	(9.4 - 8.8)	87	20	−	−	−	99	4	1.0	9.0	(9.4 - 8.6)	78	21
Insulin + 18-146	1.0	9.0	(9.2 – 8.8)	130	30	0.3	9.5	(10.6 – 8.4)	70	3	1.5	8.8	(9.1 - 8.5)	130	35
Insulin + IgG	1.2	8.9	(9.1 – 8.7)	91	21	1.8	8.7	(9.9 – 7.6)	55	2	1.7	8.8	(9.1 - 8.4)	85	23

ESM Table 7 Continued…

Treatment	S323L EC$_{50}$	S323L pEC$_{50}$	S323L 95% CI pEC$_{50}$	S323L E$_{max}$ (% Ins)	S323L E$_{max}$ (% WT Ins)	F382V EC$_{50}$	F382V pEC$_{50}$	F382V 95% CI pEC$_{50}$	F382V E$_{max}$ (% Ins)	F382V E$_{max}$ (% WT Ins)	K460E EC$_{50}$	K460E pEC$_{50}$	K460E 95% CI pEC$_{50}$	K460E E$_{max}$ (% Ins)	K460E E$_{max}$ (% WT Ins)
Insulin	>58.2	>7.2	–	100	9	1.8	8.7	(9.0 – 8.5)	100	19	1.9	8.7	(9.1 - 8.3)	100	95
18-44	–	–	–	11	1	–	–	–	0	0	–	–	–	4	4
18-146	–	–	–	0	0	–	–	–	0	0	–	–	–	66	63
IgG	–	–	–	0	0	–	–	–	5	1	–	–	–	1	1
Insulin + 18-44	59.3	7.2	(7.3 – 7.1)	267	24	1.3	8.9	(9.4 – 8.4)	95	18	1.7	8.7	(9.1 - 8.4)	106	101
Insulin + 18-146	55.8	7.2	(7.5 – 7.0)	311	28	1.1	8.9	(9.4 – 8.5)	121	23	1.8	8.7	(8.9 - 8.5)	130	124
Insulin + IgG	>97.2	>7.0	–	122	11	1.5	8.8	(9.6 – 8.0)	84	16	2.2	8.6	(8.8 - 8.5)	101	96

ESM Table 7 Continued…

Treatment	D707A EC$_{50}$	D707A pEC$_{50}$	D707A 95% CI pEC$_{50}$	D707A E$_{max}$ (% Ins)	D707A E$_{max}$ (% WT Ins)
Insulin	–	–	–	100	0
18-44	–	–	–	500	1
18-146	–	–	–	500	1
IgG	–	–	–	500	1
Insulin + 18-44	–	–	–	7500	15
Insulin + 18-146	–	–	–	1500	3
Insulin + IgG	–	–	–	1000	2

References for ESM Table 7

1. M. Rouard, F. Macari, O. Bouix, C. Lautier, J.F. Brun, P. Lefebvre, E. Renard, J. Bringer, C. Jaffiol, F. Grigorescu. Identification of two novel insulin receptor mutations, Asp59Gly and Leu62Pro, in type A syndrome of extreme insulin resistance. *Biochem. Biophys. Res. Commun.* **234**, 764-768 (1997).
2. M. Rouard, J. Bass, F. Grigorescu, T.P.J. Garrett, C.W. Ward, G. Lipkind, C. Jaffiole, D.F. Steiner, G.I. Bell, Congenital insulin resistance associated with a conformational alteration in a conserved β-sheet in the insulin receptor L1 domain, *J. Biol. Chem.* **274**, 18487-18491 (1999)
3. A.S. Alzahrani, M. Zou, E.Y. Baitei, R.S. Parhar, N. Al-Kahtani, H. Raef, A. Almahfouz, J.K. Amartey, R. Al-Rijjal, R. Hammami, B.F. Meyer, F. Al-Mohanna, Y. Shi, Molecular characterisation of a novel p.R118C mutation in the insulin receptor gene from patients with severe insulin resistance, *Clin. Endocrinol. (Oxf.)* **76**, 540-547 (2012).
4. E. Raffan, M. Soos, N. Rocha, A. Tuthill, R. Thomsen, C.S. Hyden, J.W. Gregory, P. Hindmarsh, M. Dattani, E. Cochran, J. Al Kaabi, P. Gorden, I. Basso, N. Morling, S. O'Rahilly, R.K. Semple, Founder effect in the Horn of Africa for an insulin receptor mutation that may impair receptor recycling, *Diabetologica* **54**, 1057-1065 (2011).
5. P. Carrera, R. Cordera, M. Ferrari, L. Cremonesi, R. Taramelli, G. Andraghetti, C. Carducci, N. Dozio, G. Pozza, S.I. Taylor, Subsitution of Leu for Pro-193 in the insulin receptor in a patient with a genetic form of severe insulin resistance, *Hum. Mol. Genet.* **2**, 1437-1441 (1993).
6. Y. Takata, T. Imamura, T. Haruta, K. Egawa, Y. Takada, T. Sawa, G.H. Yang, M. Kobayashi, Leu 193 mutation in the cysteine rich region of the insulin receptor inhibits the cleavage of the insulin receptor precursor but not insulin binding, *Biochem. Biophys. Res. Commun.* **203**, 763-767 (1994).
7. I. Hamer, M. Foti, R. Emkey, M. Cordier-Bussat, J. Phillippe, P. De Meyts, C. Maeder, C. Kahn, J.L. Carpentier, An argenine to cysteine 252 mutation in insulin receptors from a patient with severe insulin resistance inhibits receptor internalisation but preserves signalling events, *Diabetologia* **45**, 657-667 (2002).
8. P. Roach, Y. Zick, P. Formisano, D. Accili, S.I. Taylor, P. Gorden, A novel human insulin receptor gene mutation uniquely inhibits insulin binding without impairing posttranslational processing, *Diabetes* **43**, 1096-192 (1994)
9. A. Krook, S. Kumar, I. Laing, A.J.M Boulton, J.H. Wass, S. O'Rahilly, Molecular screening of the insulin receptor gene in syndromes of insulin resistance, *Diabetes* **43**, 357-368 (1994).
10. M. Taouis, R. Levy-Toledano, P. Roach, S. Taylor, P. Gorden, Structural basis by which a recessive mutation in the alpha-subunit of the insulin receptor affects insulin binding, *J. Biol. Chem.* **269**, 14912-14918 (1994).
11. A. Krook, M. Soos, S. Kumar, K. Siddle, S. O'Rahilly, Functional activation of mutant human insulin receptor by monoclonal antibody, *Lancet* **347**, 1586-1590 (1996).
12. D. Accili, C. Frapier, L. Mosthaf, C. McKeon, S.C. Elbein, M. Permutt, E. Ramos, E. Lander, A. Ullrich, S.I. Taylor, A mutation in the insulin receptor gene that impairs transport of the receptor to the plasma membrane and causes insulin-resistant diabetes, *EMBO J* **8**, 2509-2517 (1989).
13. D. Accili, L. Mosthaf, A. Ullrich, S.I. Taylor, A mutation in the extracellular domain of the insulin receptor impairs the ability of insulin to stimulate receptor autophosphorylation, *J. Biol. Chem.* **266**, 434- 439 (1991).
14. H. Kadowaki, T. Kadowaki, A. Cama, B. Marcus-Samuels, A. Rovira, C.L. Bevins, S.I. Taylor, Mutagenesis of lysine 460 in the human insulin receptor. Effects upon receptor recycling and cooperative interactions among binding sites, *J. Biol. Chem.* **265**, 21285-96 (1990).
15. M. Kobayashi, J.M. Olefsky, J. Elders, M.E. Mako, B.D. Given, H.K. Schwedwie, R.H. Fiser, R.L. Hintz, J.A. Horner, A.H. Rubenstein, Insulin resistance due to a defect distal to the insulin

receptor: demonstration in a patient with leprechaunism, *Proc. Natl. Acad. Sci. U.S.A* **75**, 3469-3473 (1978).

16. S.I. Taylor, J. Roth, R.M. Blizzard, M.J. Elders, Qualitative abnormalities in insulin binding in a patient with extreme insulin resistance: decreased sensitivity to alterations in temperature and pH, *Proc. Natl. Acad. Sci. U.S.A.* **78**, 7157-7161 (1981).
17. S.I. Taylor, S. Leventhal, Defect in cooperativity in insulin receptors from a patient with a congenital form of extreme insulin resistance, *J. Clin. Invest.* **71,** 1676-1685 (1983).
18. L.M. T'Hart, D. Lindhout, G.C.M. Van der Zon, H. Kayserilli, M.Y. Apak, W.J. Kleijer, E.R. Van der Vorm, J.A. Maassen, An insulin receptor mutant (Asp707 -> Ala), involved in leprechaunism, is processed and transported to the cell surface but unable to bind insulin, *J. Biol. Chem.* **271**, 18719-18724 (1996).
19. A. Krook, D.E. Moller, K. Dib, S. O'Rahilly, Two naturally occurring mutant insulin receptors phosphorylate insulin receptor substrate-1 (IRS-1) but fail to mediate the biological effects of insulin. Evidence that IRS-1 phosphorylation is not sufficient for normal insulin action, *J. Biol. Chem.* **271**, 7134-40 (1996).
20. A. Krook, J.P. Whitehead, S.P. Dobson, M.R. Griffiths, M. Ouwens, C. Baker, A.C. Hayward, S.K. Sen, J.A. Maasesen, K. Siddle, J.M. Tavare, S. O'Rahilly, Two naturally occurring insulin receptor tyrosine kinase domain mutants provide evidence that phosphoinositide 3-kinase activation alone is not sufficient for the mediation of insulin's metabolic and mitogenic effects, *J. Biol. Chem.* **272**, 30208-30214 (1997).

ESM Fig. 1

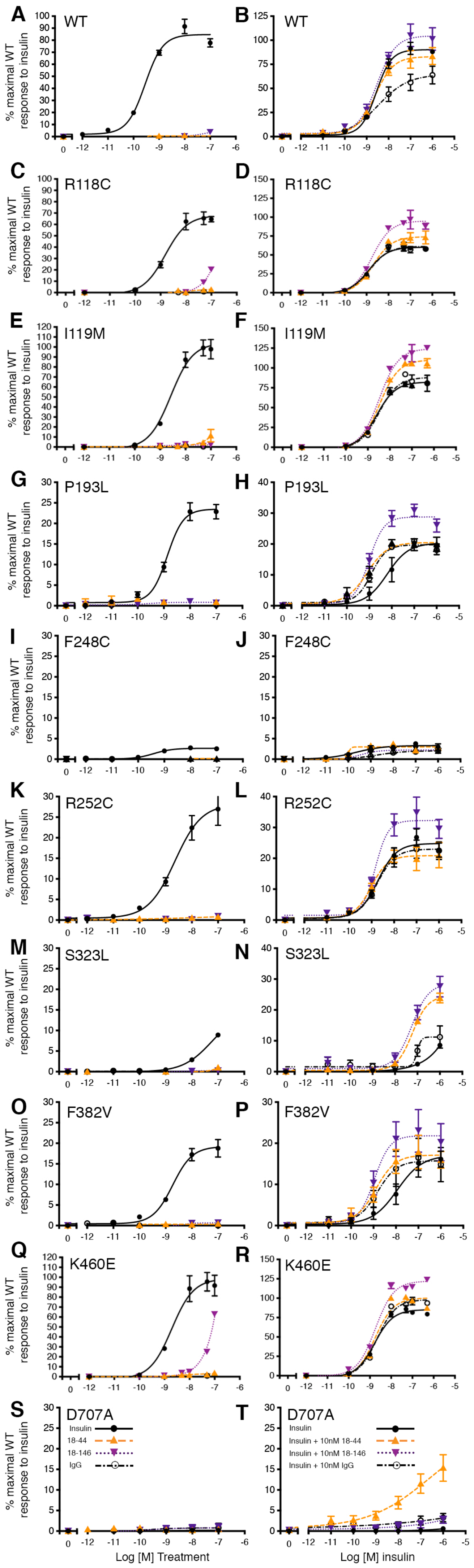
A
WT
B
WT
C
R118C
D
R118C
E
I119M
F
I119M
G
P193L
H
P193L
I
F248C
J
F248C
K
R252C
L
R252C
M
S323L
N
S323L
O
F382V
P
F382V
Q
K460E
R
K460E
S
D707A
T
D707A
% maximal WT response to insulin
Insulin
18-44
18-146
IgG
Insulin + 10nM 18-44
Insulin + 10nM 18-146
Insulin + 10nM IgG
Log [M] Treatment
Log [M] insulin

ESM Fig. 1 Insulin and antibody 18-44 and 18-146 stimulated autophosphorylation of mutant INSR. CHO FlpIN cells stably expressing either human WT (A, B) or R118C (C, D), I119M (E, F), P193L (G, H), F248C (I, J), R252C (K, L), S323L (M, N), F382V (O, P), K460E (Q, R), or D707A (S, T) mutant INSR were serum starved prior to 10 minute stimulation with increasing concentrations of either insulin, antibody (18-44, 18-146 or control IgG), or increasing concentrations of insulin in the presence of 10nM antibody. Cells were lysed and myc-tagged receptors were immunocaptured on 96-well plates and then incubated with biotin-conjugated 4G10 platinum antibody to detect phosphorylated tyrosine residues. Europium-labelled streptavidin was used to detect bound anti-phosphotyrosine antibody 4G10 by time-resolved fluorescence. The data points are the mean ± SEM of duplicate samples from three independent experiments and plotted on a logarithmic base 10 scale for the x-axis. Error bars are shown when larger than the size of the symbols. In (A, C, E, G, I, K, M, O, Q, S) single treatments are denoted by: insulin [black circles], 18-44 [orange upward pointing triangle], 18-146 [purple downward pointing triangle], control IgG [black open circles]. In (B, D, F, H, J, L, N, P, R, T) dual treatments are denoted by: insulin + 10nM 18-44 [orange upward pointing triangle], insulin + 10nM 18-146 [purple downward pointing triangle], insulin + 10nM control IgG [black open circles]. EC_{50} values are presented in ESM Table 7. As INSR autophosphorylation responses to stimulation with 18-44 and 18-146 were modest, for clarity of presentation this data was not included in the main manuscript and are discussed in the Electronic supplementary materials Results section 1.1.

ESM Fig. 2

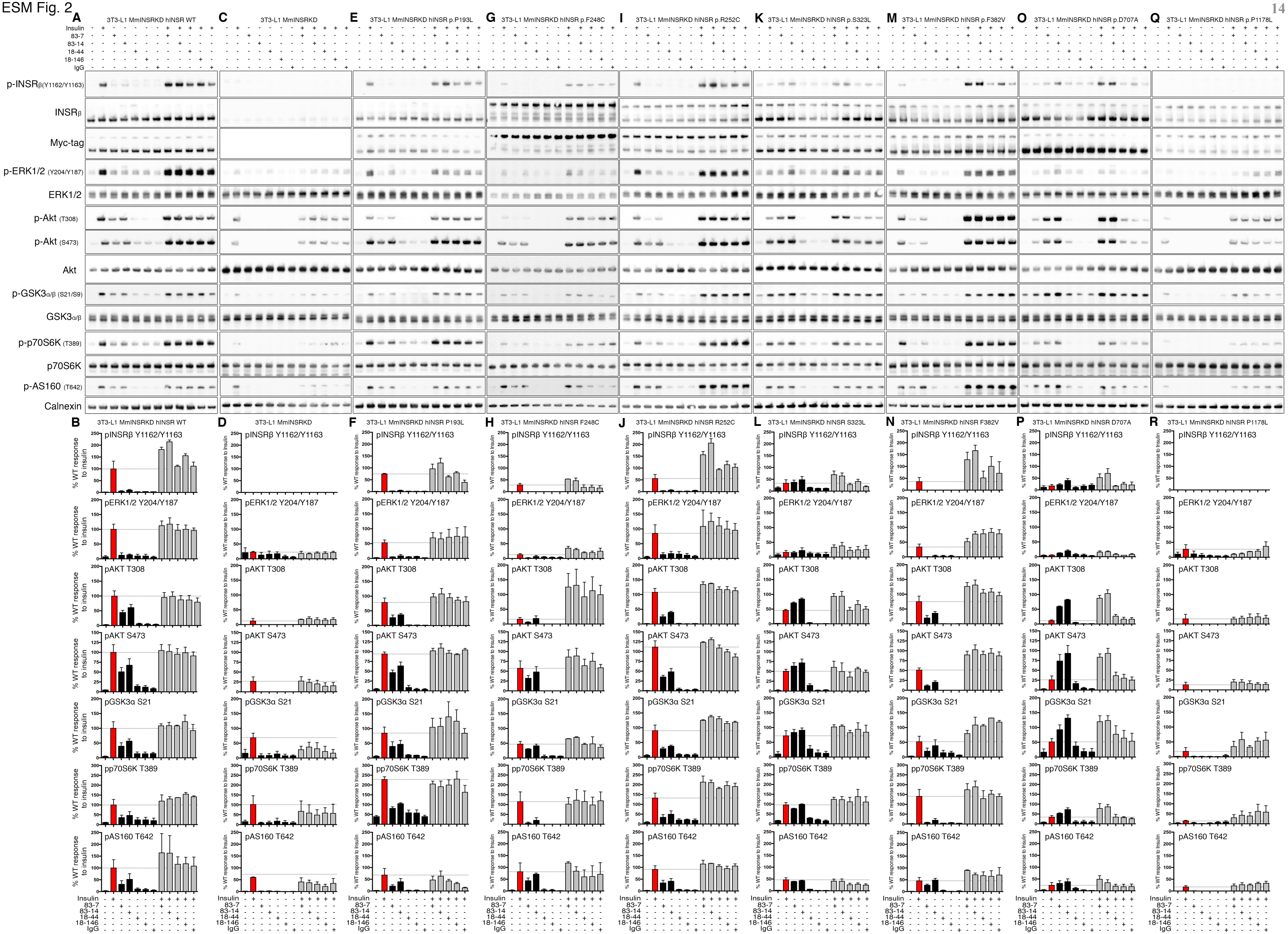
A 3T3-L1 MmINSRKD hINSR WT
C 3T3-L1 MmINSRKD
E 3T3-L1 MmINSRKD hINSR p.P193L
G 3T3-L1 MmINSRKD hINSR p.F248C
I 3T3-L1 MmINSRKD hINSR p.R252C
K 3T3-L1 MmINSRKD hINSR p.S323L
M 3T3-L1 MmINSRKD hINSR p.F382V
O 3T3-L1 MmINSRKD hINSR p.D707A
Q 3T3-L1 MmINSRKD hINSR p.P1178L
Insulin
83-7
83-14
18-44
18-146
IgG
p-INSRβ(Y1162/Y1163)
INSRβ
Myc-tag
p-ERK1/2 (Y204/Y187)
ERK1/2
p-Akt (T308)
p-Akt (S473)
Akt
p-GSK3α/β (S21/S9)
GSK3α/β
p-p70S6K (T389)
p70S6K
p-AS160 (T642)
Calnexin
B 3T3-L1 MmINSRKD hINSR WT
D 3T3-L1 MmINSRKD
F 3T3-L1 MmINSRKD hINSR P193L
H 3T3-L1 MmINSRKD hINSR F248C
J 3T3-L1 MmINSRKD hINSR R252C
L 3T3-L1 MmINSRKD hINSR S323L
N 3T3-L1 MmINSRKD hINSR F382V
P 3T3-L1 MmINSRKD hINSR D707A
R 3T3-L1 MmINSRKD hINSR P1178L
pINSRβ Y1162/Y1163
pERK1/2 Y204/Y187
pAKT T308
pAKT S473
pGSK3α S21
pp70S6K T389
pAS160 T642
% WT response to insulin

ESM Fig. 2 Activation of signalling pathways downstream of WT and mutant INSR by insulin and antibody stimulation. Figure is indicative of the full extent of the study and displays the seven mutant INSR, control 3T3-L1 MmINSRKD and 3T3-L1 MmINSRKD hINSR WT cells treated with either insulin, antibody (83-7, 83-14, 18-44 and 18-146) or insulin in the presence of antibody. 3T3-L1 MmINSRKD hINSR WT (A, B), 3T3-L1 MmINSRKD (C, D), 3T3-L1 MmINSRKD hINSR P193L (E, F), F248C (G, H), R252C (I, J), S323L (K, L), F382V (M, N), D707A (O, P), and P1178L (Q, R) adipocytes were grown in the presence of 1µg/ml DOX for 8 days prior to overnight serum-starvation on day 13 of differentiation. Adipocytes were then stimulated with either 10nM insulin, 10nM antibody (83-7, 83-14, 18-146, 18-44, control IgG) or 10nM insulin containing 10nM antibody for 10 minutes at 37°C/5%CO_2. Following stimulation, cells were washed and snap frozen prior to lysis and Western blot. Phospho-INSRβ, p-ERK1/2, p-AKT, p-GSK3α, p-p70S6K, and p-AS160 densitometry after normalisation to the mean band intensity of total INSRβ, myc-tagged INSRβ, AKT, ERK1/2, GSK3α/β, p70S6K, and calnexin for each biological replicate. Graphed data are the mean ± SEM of three independent experiments and are expressed as relative to hINSR WT response to insulin stimulation. Antibodies 18-44 and 18-146 were included in the initial screening assays of antibody-stimulated mutant INSR autophosphorylation (ESM Figure 1 & ESM Table 7), and results warranted assessing their ability to stimulate signal activation downstream of mutant INSR. However, the minor improvements in receptor autophosphorylation caused by treatment with insulin in the presence of either 18-44 or 18-146 was not observed to result in increased activation of signalling molecules downstream of the mutant INSR. As downstream signalling responses to stimulation with 18-44 and 18-146 were modest, for clarity of presentation this data was not included in the main manuscript and are discussed in the Electronic supplementary materials Results section 1.2.

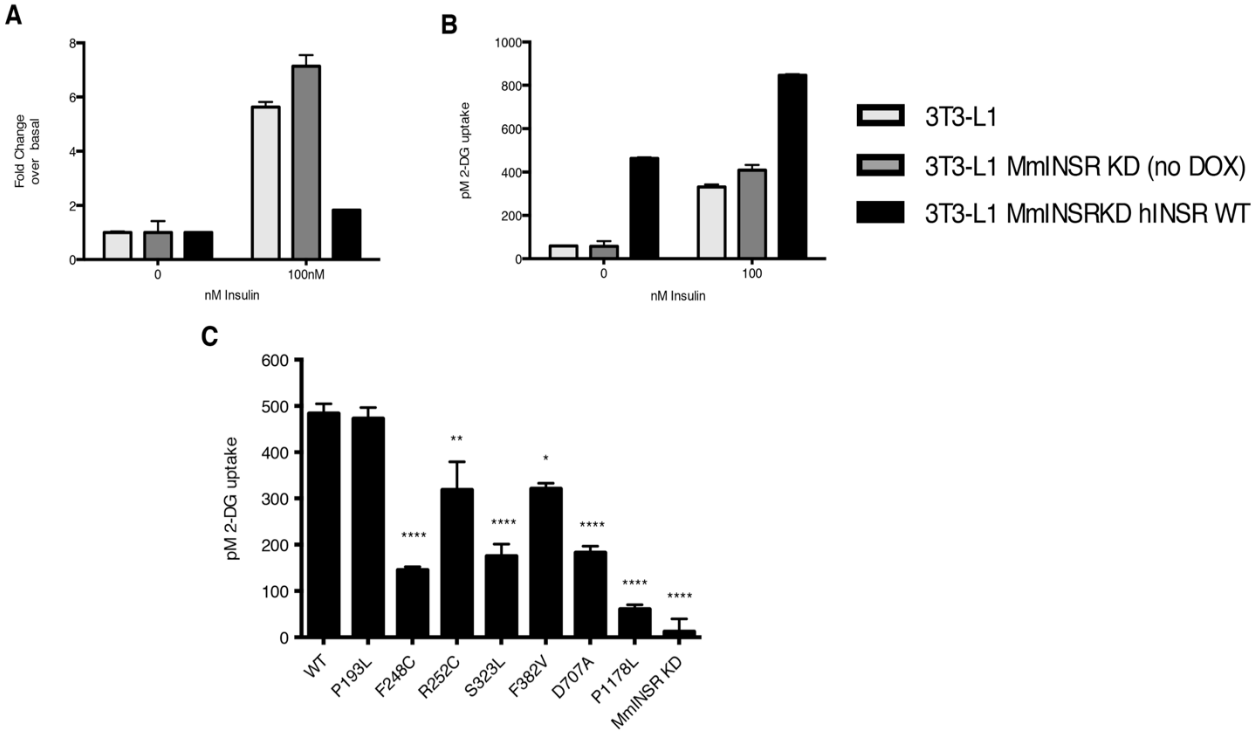

ESM Fig. 3 Basal glucose uptake differs between each of the 3T3-L1 MmINSRKD cell lines overexpressing mutant INSR. (**A** & **B**) Insulin stimulated glucose uptake in parent 3T3-L1 cells that were used to generate 3T3-L1 MmINSRKD cells, 3T3-L1 MmINSRKD cells cultured without DOX induction of knockdown of endogenous mouse INSR, and 3T3-L1 MmINSRKD hINSR WT cells cultured in the presence of DOX to induce knockdown of endogenous mouse *Insr* and expression of hINSR WT. (**C**) 3T3-L1 MmINSRKD hINSR (mutant as indicated) adipocytes were grown in the presence of 1µg/ml DOX for 10 days prior to overnight serum-starvation on day 15 of differentiation. Cells were then glucose starved for 30 minutes prior to the addition of 2-Deoxy-D-glucose for 5 minutes. Cells were then washed, lysed and assessed for 2-Deoxy-D-glucose uptake. Data is the mean ± SEM from three independent experiments. The statistical significance between non-stimulated basal 2-Deoxy-D-glucose uptake of each of the mutant receptor expressing cell lines was determined by one-way ANOVA with Tukey's multiple comparison test. Statistical significance between WT expressing cells and mutant INSR expressing cells denoted by * $p<0.05$; ** $p<0.01$, and **** $p<0.0001$.

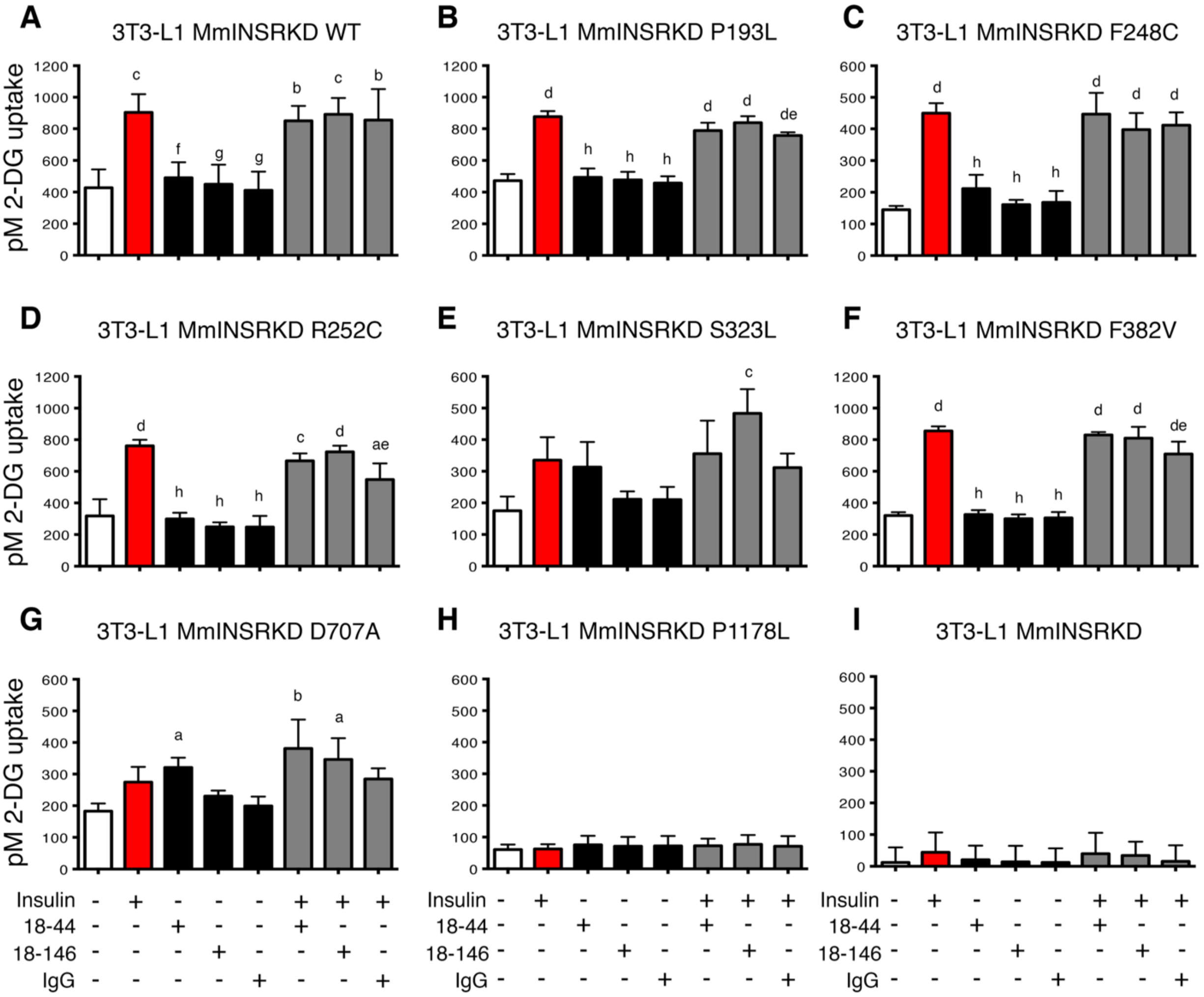

ESM Fig. 4 Insulin, antibody 18-44 and 18-146 stimulated glucose uptake via WT and mutant INSR.

3T3-L1 MmINSRKD hINSR (mutant as indicated) adipocytes were grown in the presence of 1µg/ml DOX for 10 days prior to overnight serum-starvation on day 15 of differentiation. The cells were stimulated for 30 minutes with either 10nM insulin, 10nM antibody (18-44, 18-146, control IgG) or 10nM insulin containing 10nM antibody prior to the addition of 2-Deoxy-D-glucose for 5 minutes. Cells were then washed, lysed and assessed for 2-Deoxy-D-glucose uptake. Data is the mean ± SD from three independent experiments, statistical significance determined by one-way-ANOVA with Tukey's multiple comparison test. Statistical significance from un-stimulated basal is denoted by a, b, c, d ($p<0.05$, $p<0.01$, $p<0.001$, $p<0.0001$ respectively). Statistical significance from 10nM insulin treatment is denoted by e, f, g, h ($p<0.05$, $p<0.01$, $p<0.001$, $p<0.0001$ respectively). Statistical significance from 10nM IgG control treatment is denoted by i, j, k, l. Statistical significance from 10nM insulin in the presence of 10nM IgG control is denoted by m, n, o ($p<0.05$, $p<0.01$, $p<0.001$ respectively). As glucose uptake responses to stimulation with 18-44 and 18-146 were modest, for clarity of presentation this data was not included in the main manuscript and are discussed in the Electronic supplementary materials Results section 1.3.
